# Histone acetyltransferase TaHAG1 acts as a crucial regulator to strengthen salt tolerance of hexaploid wheat

**DOI:** 10.1093/plphys/kiab187

**Published:** 2021-04-23

**Authors:** Mei Zheng, Jingchen Lin, Xingbei Liu, Wei Chu, Jinpeng Li, Yujiao Gao, Kexin An, Wanjun Song, Mingming Xin, Yingyin Yao, Huiru Peng, Zhongfu Ni, Qixin Sun, Zhaorong Hu

**Affiliations:** State Key Laboratory for Agrobiotechnology/Key Laboratory of Crop Heterosis and Utilization (MOE)/Beijing Key Laboratory of Crop Genetic Improvement, China Agricultural University, Beijing 100193, PR China

## Abstract

Polyploidy occurs prevalently and plays an important role during plant speciation and evolution. This phenomenon suggests polyploidy could develop novel features that enable them to adapt wider range of environmental conditions compared with diploid progenitors. Bread wheat (*Triticum aestivum* L., BBAADD) is a typical allohexaploid species and generally exhibits greater salt tolerance than its tetraploid wheat progenitor (BBAA). However, little is known about the underlying molecular basis and the regulatory pathway of this trait. Here, we show that the histone acetyltransferase TaHAG1 acts as a crucial regulator to strengthen salt tolerance of hexaploid wheat. Salinity-induced *TaHAG1* expression was associated with tolerance variation in polyploidy wheat. Overexpression, silencing, and CRISPR-mediated knockout of *TaHAG1* validated the role of *TaHAG1* in salinity tolerance of wheat. TaHAG1 contributed to salt tolerance by modulating reactive oxygen species (ROS) production and signal specificity. Moreover, TaHAG1 directly targeted a subset of genes that are responsible for hydrogen peroxide production, and enrichment of TaHAG1 triggered increased H3 acetylation and transcriptional upregulation of these loci under salt stress. In addition, we found the salinity-induced TaHAG1-mediated ROS production pathway is involved in salt tolerance difference of wheat accessions with varying ploidy. Our findings provide insight into the molecular mechanism of how an epigenetic regulatory factor facilitates adaptability of polyploidy wheat and highlights this epigenetic modulator as a strategy for salt tolerance breeding in bread wheat.

## Teaser

A histone acetyltransferase contributes to salt tolerance by mediating reactive oxygen species production in hexaploid wheat, which is involved in salt stress adaptability of polyploidy wheat.

## Introduction

Polyploidy or whole-genome duplication (WGD) plays a major role in shaping genome evolution and speciation of flowering plants, including many important crops such as wheat (*Triticum aestivum* L.), cotton (*Gossypium hirsutum*), and canola (*Brassica napus*; Leitch and Leitch 2008; Madlung, 2013; Soltis and Soltis, 2016). The prevalent occurrence of polyploidy in the evolutionary history of plants suggests polyploid organisms might have better adaptability to a wider range of environmental conditions than their diploid progenitors (Adams and Wendel, 2005; Otto, 2007; Jiao et al., 2011; Van de Peer et al., 2017; Wu et al., 2020). Compared with diploid plants, polyploid plants usually develop favorable physiological traits such as large organs, increased photosynthetic capacity, and enhanced tolerance to biotic and abiotic stresses, which can be immediately reflected upon WGD or emerged during evolution (Ni et al., 2009; Chao et al., 2013; Chalhoub et al., 2014; Yang et al., 2014; Zhang et al., 2015). The intricate mechanisms underlying evolutionary novelty and adaptability to diverse environments of polyploidy need to be disentangled and characterized to effectively manipulate the trait in breeding. Recent advances have shown that both genetic and epigenetic changes involved in the expression of homeologs and phenotypic variation that may facilitate adaptive evolution in polyploid plants (Wendel et al., 2016; Van de Peer et al., 2017; Ding and Chen, 2018; Jiao et al., 2018). However, our understanding of the regulatory pathway involved in stress adaptability of polyploidy is largely limited in major crops.

As one of the major staple crop worldwide, bread wheat is a typical allohexaploid species which combines the D genome from *Aegilops tauschii* with the AB genomes from tetraploid wheat (Berkman et al., 2013). Hexaploid bread wheat has better adaptability to adverse environmental conditions compared with tetraploid wheat (Dubcovsky and Dvorak, 2007; Li et al., 2015). For instance, it is well acknowledged that hexaploid bread wheat generally exhibits more salt tolerant than its tetraploid progenitor *T*riticum *turgidum* (Dvorak et al., 1994; Munns et al., 2012; Yang et al., 2014). Further studies indicated high-affinity potassium (K^+^) transporter *TaHKT1;5-D* from chromosome 4DL that encodes a Na^+^-selective transporter, could be one of the candidates for salinity tolerance in bread wheat via shoot Na^+^ exclusion (Byrt et al., 2014). Nevertheless, salt resistance is a complex trait, the underlying molecular basis of salt tolerance in wheat and mechanisms involved in tolerance variation between hexaploid and tetraploid wheats still should be further explored.

Reactive oxygen species (ROS) are highly reactive molecules and generally induced as part of the response to environmental constraints such as salinity and drought stresses (Miller et al., 2010). Excess production of ROS is potentially deleterious for tissues, ultimately disrupt the cell redox state commonly referred to as “oxidative stress” that may even lead to cell death (Apel and Hirt, 2004; Marino et al., 2012). Nevertheless, growing number of studies indicate ROS are not exclusively noxious but also act as key components in a wide range of biological processes in plants, such as root hair growth, stomatal closure, response to abiotic, and/or biotic stresses (Marino et al., 2012; Liu et al., 2014; Waszczak et al., 2018; Zhang et al., 2020). In plants, respiratory burst oxidase homologs (Rbohs) have been proposed for the fine-tuning of ROS production and signal specificity (Torres and Dangl, 2005; Ma et al., 2012). However, the mechanism details of their transcriptional regulation and contribution to salt tolerance in polyploidy wheats remain elusive.

Epigenetic regulatory mechanisms, including DNA/RNA methylation, histone modification, and chromatin remodeling, play important roles in plant development and in response to environmental changes (Kinoshita and Seki, 2014; Luo et al., 2017; Cheng et al., 2018; Chang et al., 2020). Among these modifications, histone acetylation has been extensively studied and mainly occurs in lysine residues on histone tails. Histone acetylation can be established and removed by corresponding enzymes, providing dynamic mechanisms for transcriptional regulation (Perduns et al., 2015; Hou et al., 2017; Zhou et al., 2017; Kim et al., 2018). Increasing studies suggested histone acetylation also acts as new layer of regulation to cope with abiotic environmental stress through modulation of key regulatory factors (Sako et al., 2016; Zheng et al., 2016, 2019; Li et al., 2017; Ueda et al., 2017; Li et al., 2019). However, little is known about the involvement of histone acetylation in salt tolerance regulation of polyploidy wheat.

In this study, we showed that both synthetic and natural hexaploid wheats exhibit superior salt tolerance relative to the tetraploid wheat with major physiological and biochemical properties. We identified a histone acetyltransferase TaHAG1 is involved in salinity tolerance variation in polyploidy wheat. Overexpression of *TaHAG1* enhances salt stress tolerance, whereas RNAi or CRISPR-mediated knockout of *TaHAG1* causes strong sensitivity to salt stress in wheat. Importantly, TaHAG1 directly targets a subset of genes involved in ROS production and triggers increased H3 acetylation and transcriptional upregulation of these loci in response to salt stress. According to our results, the TaHAG1-mediated ROS production and homeostasis is involved in salt stress adaptability of polyploidy wheat. These results revealed an epigenetic mechanism of a histone acetyltransferase confers salt tolerance in allohexaploid wheat, making this gene a potential target for salt tolerance improvement in wheat as well as in other crops.

## Results

### Hexaploid wheats exhibit enhanced salinity tolerance compared to tetraploid progenitors

**Figure 1:**
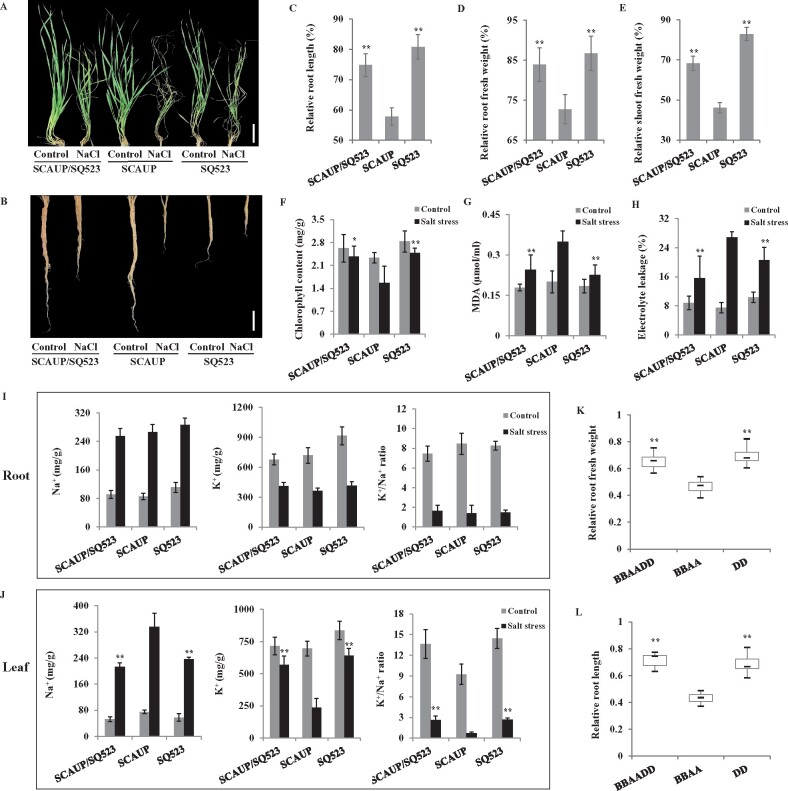
Effects of salt stress on allohexaploid wheats, allotetraploid wheat, *T. turgidum* and diploid species *Ae. tauschii.* A and B, Phenotype of 4-week-old synthetic allohexaploid wheat (SCAUP/SQ523), its tetraploid parent SCAUP and its diploid parent SQ523 under control or 200 mM NaCl, shoot (A); root (B). Scale bars: shoot, 5 cm; root, 5 cm. C–E, Comparison of the seedlings growth of 4-week-old SCAUP/SQ523, SCAUP, and SQ523 under salt stress conditions. Root length (C), root fresh weight (D), and shoot fresh weight (E). The *y*-axis denotes a percentage of NaCl-treated seedlings relative to the same genotype grown on control conditions. F–H, Comparison of chlorophyll content (F), MDA content (G), and relative electrolyte leakage (H) of leaves from SCAUP/SQ523, SCAUP, and SQ523 plants after exposure to 200 mM NaCl. The percentage of electrolyte leakage was determined as the percentage of the conductivity before boiling and after boiling of the detached plants. I and J, Changes in Na^+^ contents, K^+^ contents, and K^+^ to Na^+^ ratios in root (I) and leaf (J) of SCAUP/SQ523, SCAUP, and SQ523 plants before and under salt stress conditions. In (C–J), mean and standard deviation (sd) values were derived from measurements of at least 15 seedlings from three independent assays. Asterisks indicate significant differences between SCAUP/SQ523, SQ523 plants with SCAUP plants under salt stress conditions. Statistical significance was determined by a two-sided *t* test: *P < 0.05, **P < 0.01. K and L, Comparative analyses of the relative root weight (K) and roots length (L) for natural wheat accessions with varying ploidy under salt stress. Box edges represent the 0.25 quantile and 0.75 with the maximum, minimum, and average values are represented by bold lines. Differences between the varying ploidy (*n* = 16) were analyzed by Student’s *t* test. **P < 0.01.

To assess the salt tolerance difference after the formation of allopolyploid wheat, a synthetic allohexaploid wheat (SCAUP/SQ523, BBAADD) with its allotetraploid (SCAUP, BBAA) and diploid *Ae. tauschii* (SQ523, DD) parents were subjected to salt stress treatment. We recorded the growth behavior at specific time points and quantified root length, root and shoot fresh weight, and a range of physiological indexes for each of the three genotypes under normal and salt stress conditions. The SCAUP/SQ523 and SQ523 showed more tolerance than SCAUP under salt stress (Figure 1, A and B). The SCAUP/SQ523 exhibited a less severe phenotype in root elongation under salt stress, >75% of the control. In contrast, the maximum root lengths in SCAUP under salt stress were ˂60% of the controls (Figure 1, B and C). Moreover, the total biomass including both roots and shoots fresh weight in SCAUP/SQ523 under salt stress was greater than SCAUP, but did not differ statistically from the SCAUP in control conditions (Fig 1, D and E). In addition, associated with growth vigor, SCAUP/SQ523, and SQ523 showed reduced cell membrane damage, reduced MDA content, and enhanced chlorophyll content compared with SCAUP plants under salt stress (Figure 1, F–H).

As low Na^+^ and high K^+^/Na^+^ ratio in the cytoplasm are pivotal for salt tolerance, we further examined the Na^+^ content and K^+^/Na^+^ ratio for each of the three genotypes. As shown in Figure 1, I and J, Na^+^ content and K^+^/Na^+^ ratio were similar in roots of SCAUP/SQ523, SCAUP, and SQ523 plants under both normal and salt stress conditions (Figure 1I). However, in leaves, the SCAUP/SQ523 and SQ523 showed much lower Na^+^ content and higher K^+^/Na^+^ ratios than SCAUP under salt stress (Figure 1J). Notably, the diploid *Ae. tauschii* SQ523 showed a higher fitness under salt stress including higher growth vigor and K^+^/Na^+^ ratio, suggesting that the D subgenome with a major contribution to stronger salt tolerance in synthetic allohexaploid wheat.

Next, we assessed the salt tolerance difference of 48 natural wheat accessions with varying ploidy, including 16 allohexaploid wheats (BBAADD), 16 allotetraploid (BBAA), and 16 diploid species *Ae. tauschii* (DD). These genetic resources are collected from Asia, Europe, Middle East, and North America including landraces, modern cultivars, and wild species (Supplemental Table S1). The change ratio of root length, root, and shoot fresh weight before and after salt treatment was used as the indicator to evaluate the salt tolerance. Across the three different ploidy levels, NaCl had a much stronger growth inhibition on allotetraploid seedlings than allohexaploid wheats and *Ae. Tauschii*. The average ratio of roots length, roots and shoots fresh weight of allohexaploid wheats and *Ae. tauschii* were significantly higher than that of the allotetraploid groups (Figure 1, K and L; Supplemental Figure S1). Together, consistent with previous studies (Dubcovsky et al., 1996; Munns et al., 2012; Yang et al., 2014), our results also indicated the hexaploid wheat and its diploid ancestral species *Ae. tauschii* showed better fitness than tetraploid wheat when subjected to salt stress.

### Salinity induced *TaHAG1* expression was associated with salt tolerance variation in polyploidy wheat

To explore the possible molecular basis for salt tolerance difference of polyploidy wheat, we first profiled the transcriptome analysis of SCAUP/SQ523 with its parents SCAUP and SQ523 under normal and salt stress conditions. A total of 1,725 genes were significantly upregulated (by at least two-fold, FDR < 0.01) both in SCAUP/SQ523 and SQ523 after salt treatment; however, only 249 genes were upregulated both in SCAUP/SQ523 and SCAUP under salt stress (Supplemental Table S2; Supplemental Figure S2). The more overlapping genes between SCAUP/SQ523 and SQ523 combining with the physiological and morphological properties under salt stress, suggesting synthetic hexaploid wheats resembled more closely to the diploid parent than the allotetraploid parent under salt stress treatment. Among these upregulated transcripts, one gene *TraesCS1D02G134200* showed significant upregulation both in SCAUP/SQ523 and SQ523 in response to salt stress, but the elevated expression of its homeologs was limited in SCAUP under salt treatment (Supplemental Table S2). Sequence alignment indicated it encodes a histone acetyltransferase, a putative ortholog of AtHAG1/GCN5 in Arabidopsis and OsHAG702 in rice. We thus named it *TaHAG1*. Phylogenetic analysis indicated TaHAG1 belongs to the GNAT subfamily of type-A HATs that comprised members including BdHAG1 in *Brachypodium distachyon*, OsHAG702 in rice, ZmGNAT101 in maize, and AtHAG1/GCN5 in Arabidopsis (Supplemental Figure S3A). In our previous study, the constitutive expression of this gene in the Arabidopsis *hag1*/*gcn5* mutant can complement its sensitive phenotype to salt stress (Zheng et al., 2019). A BLAST search against the Zang1817 genome and 10+ wheat genome databases revealed there are three *TaHAG1* homeologs in hexaploid wheat, designated as *TaHAG1-A*, *TaHAG1-B*, and *TaHAG1-D*, respectively, according to their chromosome location (Supplemental Figure S3B; Guo et al., 2020; Walkowiak et al., 2020). Three *TaHAG1* homeologs showed consistent intron/exon organization and high similarity in coding sequences, which can only be distinguished from one another by virtue of single nucleotide polymorphisms. All three *TaHAG1* homeologs encode 507 amino acids and share high similarity (99.8%), which contain conserved N-terminal HAT domain and C-terminal bromodomain, suggesting TaHAG1 plays both a writer and a reader of histone acetylation (Supplemental Figure S3C).

**Figure 2:**
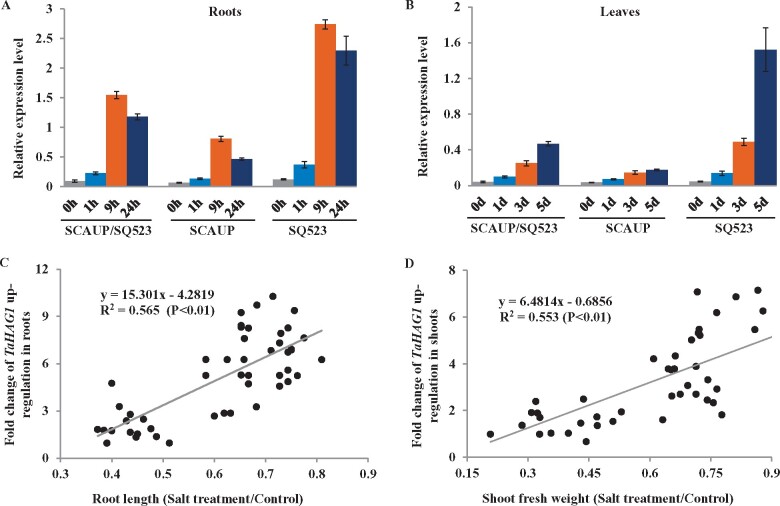
Salinity-induced *TaHAG1* expression was correlated with salt tolerance variation in polyploidy wheat. A and B, Expression patterns of the *TaHAG1* gene in roots (A) and leaves (B) of synthetic allopolyploid wheat SCAUP/SQ523 and their parents SCAUP, SQ523 under salt stress. Seven-day-old seedlings grown in a hydroponic culture were treated with 200 mM NaCl for the indicated times. The expression of *β-Actin* was used to normalize mRNA levels. The values are means (±se) of three biological replicates. C and D, Correlation coefficients between the relative roots length (C), relative aboveground fresh weight (D) with the expression levels of *TaHAG1* among different ploidy wheat accessions. The *x*-axis denotes a percentage of NaCl-treated seedlings relative to the same genotype grown on control condition. The *y*-axis denotes the fold change of *TaHAG1* upregulation expression in root or shoot of wheat accessions with different ploidy before and after 200 mM NaCl treatment. Statistical significance was determined by ANOVA.

We further examined the expression of *TaHAG1* in SCAUP/SQ523 with SCAUP and SQ523 under salt stress. In roots, the transcript of *TaHAG1* increased in 1 h after salt treatment and peaked at 9 h, then slightly declined; but the mRNA increased slowly in SCAUP and substantially lower than in SCAUP/SQ523 and SQ523 for all indicated time (Figure 2A;Supplemental Figure S4A). In leaves, the transcript of *TaHAG1* gradually increased following the salt treatment (Figure 2B). Remarkably, the expression of *TaHAG1* was also substantially higher in SCAUP/SQ523 and SQ523 than SCAUP for all the indicated time (Figure 2B;Supplemental Figure S4B).

We further examined whether the expression pattern of *TaHAG1* is associated with variation of salt tolerance in natural wheat accessions with varying ploidy. The expression changes of *TaHAG1* were examined in roots and leaves of 48 natural wheat accessions under salt stress, respectively. The results showed that under salt stress, the upregulation expression of *TaHAG1*, both in roots and leaves, was positively correlated with salt tolerance in wheat accessions with different ploidy (Figure 2, C and D; Figure S4C). These results suggested higher expression of *TaHAG1* was positively associated with variation of salt tolerance in polyploid wheat.

### Overexpression and knock-down of *TaHAG1* validate the positive role of *TaHAG1* in salinity tolerance of wheat

**Figure 3:**
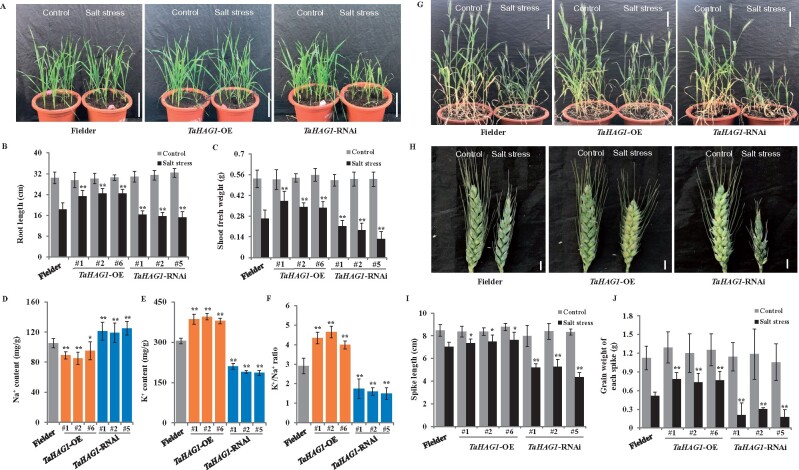
*TaHAG1* is responsible for salt tolerance in wheat. A, Phenotype of 4-week-old *TaHAG1*-OE, *TaHAG1*-RNAi lines with WT Fielder plants under normal conditions or 200 mM NaCl treatment. Scale bars: 10 cm. B and C, Comparison of root length (B) and shoot fresh weight (C) of 4-week-old *TaHAG1*-OE, *TaHAG1*-RNAi plants under salt stress conditions. D–F, Comparison of Na^+^ contents (D), K^+^ contents (E), and K^+^ to Na^+^ ratios (F) of leaves from *TaHAG1*-OE, *TaHAG1*-RNAi lines, and WT plants under salt stress conditions. G and H, Morphological phenotype of aboveground traits (G) and spikes (H) of *TaHAG1*-OE, *TaHAG1*-RNAi lines with WT Fielder plants at grain filling stage under a duration of NaCl treatments. Scale bars: Whole plants, 10 cm; spike, 1 cm. I and J, Statistical data for spike length (I) and grain weight per spike (J) of the *TaHAG1*-OE, *TaHAG1*-RNAi lines with WT Fielder plants under salt stress treatments. Mean and sd values were derived from measurements of at least 15 plants of each kind of genotype in three independent experiments. Asterisks indicate significant differences between *TaHAG1* transgenic lines with WT plants under salt stress conditions (*P < 0.05, **P < 0.01 by two-sided *t* test).

To further determine the functions of *TaHAG1* in the regulation of wheat salt resistance, we generated transgenic wheat plants that either had the *TaHAG1* overexpressed (*TaHAG1*-OE) or silenced via RNA interference (*TaHAG1*-RNAi). Ten putative *TaHAG1*-OE lines in the wheat cultivar Fielder were obtained and three *TaHAG1-*OE lines (#1, #2, and #6) with significantly elevated expression of *TaHAG1* were selected for further study (Supplemental Figure S5A). Furthermore, we generated eight *TaHAG1*-RNAi lines in Fielder background, and *TaHAG1-*RNAi lines #1, #2, and #5 showed clearly reduced *TaHAG1* expression ∼40% of wild-type (WT) Fielder (Supplemental Figure S5B). To explore salt stress tolerance conferred by the expression change of *TaHAG1*, the seedlings of *TaHAG1*-OE and *TaHAG1*-RNAi lines were subjected to salt stress together with WT plants. Under normal conditions, no obvious phenotypic variation or developmental abnormalities are observed between *TaHAG1*-OE lines, *TaHAG1*-RNAi lines, and WT plants (Figure 3A;Supplemental Figure S5C). Under salt stress treatment, both the WT plants and transgenic lines display a certain degree of growth inhibition. However, the *TaHAG1-*OE lines exhibit a less severe phenotype as compared to WT plants. In contrast, the growth is more significantly inhibited in *TaHAG1*-RNAi lines than in the WT (Figure 3A;Supplemental Figure S5C). The salt-treated root lengths are >75% of the nontreated controls in *TaHAG1*-OE lines, where salt-treated WT seedlings were ˂60% of the nontreated controls and *TaHAG1*-RNAi lines are ˂48% of the nontreated controls (Figure 3B;Supplemental Figure S5C). In addition, shoot and root fresh weight in the *TaHAG1*-OE lines also show advantages than WT, whereas *TaHAG1*-RNAi lines are more sensitive to salt stress as compared to WT plants (Figure 3C;Supplemental Figure S5D). We also compared the Na^+^, K^+^ content, and K^+^/Na^+^ ratio for leaves of WT and various transgenic lines after salt treatment. As shown in Figure 3, D–F, the *TaHAG1*-OE lines keep more K^+^, less Na^+^ content, and higher K^+^/Na^+^ ratio than WT and *TaHAG1*-RNAi lines.

We also performed salt stress treatment of *TaHAG1*-OE and *TaHAG1*-RNAi lines with WT plants in vegetative and reproductive stages of wheat growth (Figure 3G). Under normal conditions, there was no detectable difference between transgenic lines and WT plants. However, under salt stress treatments, the *TaHAG1-*OE lines show much higher salt tolerance than the WT, including higher plant height, spike length, kernel number of spike, and yield. In contrast, *TaHAG1*-RNAi lines exhibit severe growth inhibition compared to WT plants in terms of these traits (Figure 3G–J; Supplemental Figure S5, E and F).

**Figure 4:**
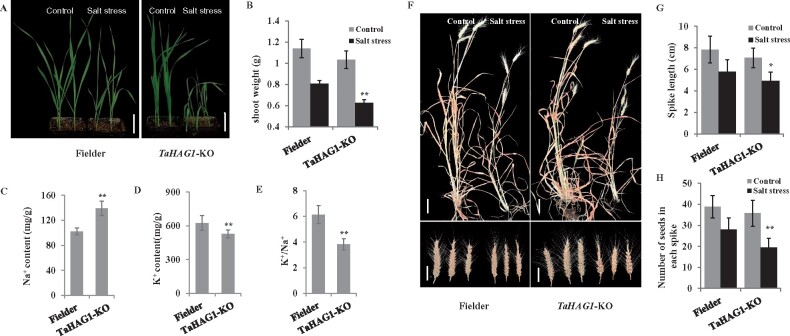
knockout of *TaHAG1* causes reduced salt tolerance in wheat. A and B, Representative phenotype (A) and statistical comparisons of shoot weight (B) of 3-week-old *TaHAG1*-KO lines with WT Fielder plants under normal conditions or 200 mM NaCl treatment. Scale bars: 5 cm. C–E, Comparison of Na^+^ contents (C), K^+^ contents (D), and K^+^ to Na^+^ ratios (E) of leaves from *TaHAG1*-KO lines and WT plants under salt stress conditions. F, Morphological phenotype of aboveground traits and spikes of *TaHAG1*-KO lines with WT Fielder plants at maturity period under a duration of NaCl treatments. Scale bars: Whole plants, 10 cm; spike, 5 cm. G and H, Statistical data for spike length (G) and number of seeds per spike (H) of the *TaHAG1*-KO lines with WT Fielder plants under salt stress. Mean and sd values were derived from measurements of at least 15 plants of each kind of genotype in three independent experiments. Asterisks indicate significant differences between *TaHAG1*-KO lines with WT plants under salt stress conditions (*P < 0.05, **P < 0.01 by two-sided *t* test).

To further validate the functions of *TaHAG1* in salt tolerance, we obtained the knockout lines of *TaHAG1* based on CRISPR/Cas9 system. The guide RNA was designed to target a highly conserved region in the first exon (Supplemental Figure S6A). We did not get the homozygous mutant with simultaneously knock out all three *TaHAG1* homeologs based on sequencing analyses of 106 *TaHAG1*-KO T_2_ plants, suggesting the homozygous mutation in three homeologs of *TaHAG1* may be lethal for wheat. So the simultaneous homozygous mutations at the two *TaHAG1* homeologs (*TaHAG1*-KO-AB) were identified and selected for further study. Sequencing analyses showed that homozygous mutant lines of *TaHAG1*-KO-AB, which confer a 1 bp insertion in *TaHAG1-A* and 25 bp deletion in *TaHAG1-B*, respectively, causing frameshifting and truncation of *TaHAG1-A* and *TaHAG1-B* homeologs (Supplemental Figure S6B). There was no obvious phenotypic variation between WT and mutant plants grown in control soil (Figure 4A;Supplemental Figure S6C). However, under salt stress treatment, the *TaHAG1-*KO lines exhibit more severe inhibits as compared to WT plants, including reduced root length and fresh weight, more chlorotic leaves and more Na^+^ content, and lower K^+^/Na^+^ ratio than WT plants (Figure 4, B–E; Supplemental Figure S6, C and D). The response of *TaHAG1*-KO lines to salt stress in vegetative and reproductive stages was also examined. As expected, the *TaHAG1-*KO lines exhibit more serious phenotypes, with significant reduced spike length, kernel number of spike, and grain yield compared to WT plants under salt stress (Figure 4, F–H; Supplemental Figure S6E). Taken together, these results of the overexpression, RNAi, and CRISPR-mediated knockout experiments strongly suggest that the *TaHAG1* gene functions as a positive regulator to salt tolerance in wheat.

### TaHAG1 contributes to salt tolerance by influencing ROS production in wheat

**Figure 5:**
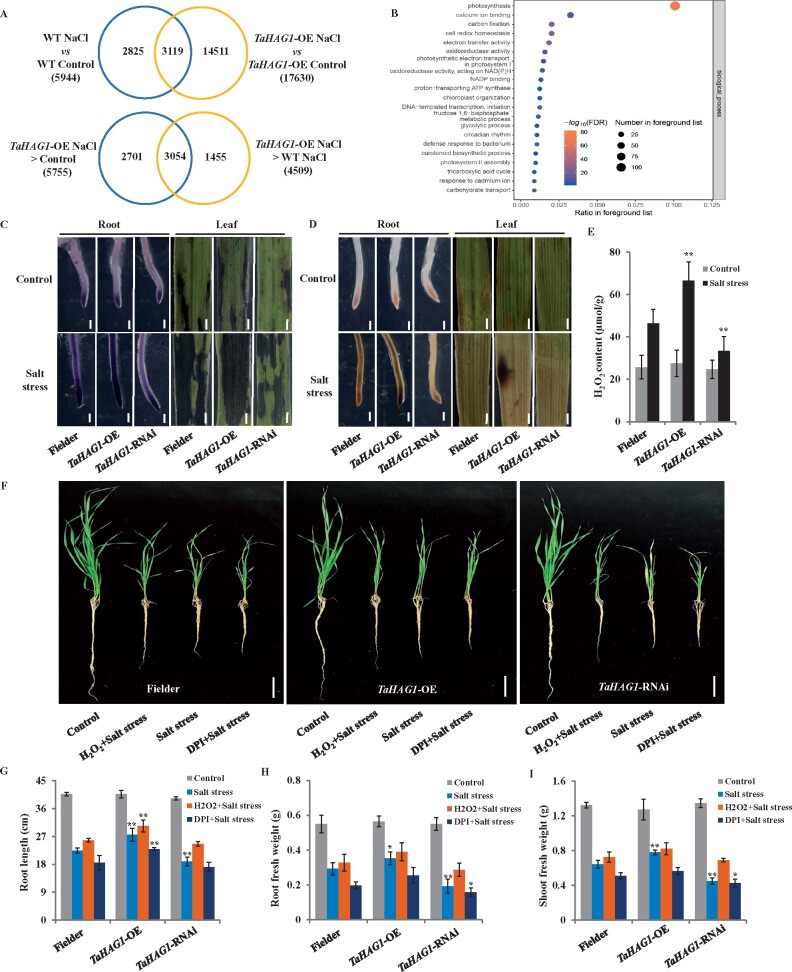
TaHAG1 mediates ROS production under salt stress. A, Venn diagrams indicating the genes that significantly upregulated in *TaHAG1*-OE plants than WT Fielder after salt stress treatment, representing potential TaHAG1 targets that are also involved in response to salt stress. B, GO enrichment analyses of the differentially expressed genes whose expression was upregulated significantly after salt stress treatment in *TaHAG1*-OE plants. C and D, Histochemical staining of NaCl-induced ROS production in roots and leaves of WT Fielder, *TaHAG1*-OE, and *TaHAG1*-RNAi lines under control or NaCl treatment visualized using NBT (C) or DAB staining (D). Dark or tawny shading indicates accumulation of H_2_O_2_. Scale bars: root, 500 μm; leaf, 1 mm. E, H_2_O_2_ contents in the seedling of the WT Fielder and *TaHAG1*-OE, *TaHAG1*-RNAi plants under control and salt stress conditions. In (C–E), 7-d-old seedlings grown in a hydroponic culture were treated with 200 mM NaCl for 9 h in roots and 48 h in leaves. F, Effects of exogenous H_2_O_2_ and DPI on salt resistance of WT Fielder, *TaHAG1*-OE, *TaHAG1*-RNAi plants. Non-stressed or stressed 1-week-old seedlings grown for 3 weeks and then photographed and quantitated. Scale bar = 5 cm. Salt stress: 200 mM NaCl; H_2_O_2_ + Salt stress: 10 mM H_2_O_2_ together with 200 mM NaCl; DPI + Salt stress: pretreatment with 10 μM DPI prior to 200 mM NaCl treatment. G–I, Comparison of the seedlings growth of WT Fielder and *TaHAG1*-OE, *TaHAG1*-RNAi plants in corresponding treatment in (F). Root length (G), root fresh weight (H), and shoot fresh weight (I). Mean and sd values were derived from measurements of at least 15 individuals of each kind of genotype. Similar results were obtained from three independent biological experiments. Asterisks indicate significant differences between *TaHAG1* transgenic lines with WT plants under the same treatment (*P < 0.05, **P < 0.01 by two-sided *t* test).

To explore the underlying molecular basis of the TaHAG1 in the regulation of wheat salt tolerance, we performed RNA sequencing (RNA-seq) to compare the transcriptomes of *TaHAG1-*OE lines and WT plants in control or salt stress conditions. Following salt stress treatment, 5,944 genes were differentially expressed (fold change >2 and FDR < 0.01) in the WT relative to control conditions (Figure 5A;Supplemental Table S3). However, NaCl treatment caused a dramatic transcriptomic change in *TaHAG1*-OE plants relative to the WT plants, with 17,630 differentially expressed genes (fold change >2 and FDR < 0.01; Figure 5A;Supplemental Table S3). This suggests that *TaHAG1*-OE plants are more active response to NaCl treatment than the WT plants in terms of transcriptomic changes. As histone acetylation is generally associated with active chromatin and enhances gene transcription (Shen et al., 2015), we mainly focus on the upregulated genes that are affected by *TaHAG1* in response to salt stress. Under NaCl treatment, 5,755 genes were significantly upregulated (fold change >2 and FDR < 0.01) in *TaHAG1-*OE lines relative to control conditions, designated *TaHAG1-*OE NaCl > Control. Moreover, 4,509 genes were upregulated (fold change >2 and FDR < 0.01) in the *TaHAG1-*OE lines compared with WT plants under salt treatment, designated *TaHAG1-*OE NaCl > WT NaCl (Figure 5A;Supplemental Table S3). We reasoned that *TaHAG1*-regulated genes involved in salt tolerance would be enriched in the overlapping 3,054 genes between *TaHAG1-*OE NaCl > Control and *TaHAG1-*OE NaCl > WT NaCl (Figure 5A;Supplemental Table S4). A further two-way analysis of variance (ANOVA)  quantified the effects of the TaHAG1 under salt stress treatment and 2,332 genes from 3,054 genes were identified with significance (*P* < 0.01). Biological pathways of photosynthesis, carbon fixation, and electron transfer activity were greatly enriched among these 2,332 genes. Moreover, gene ontology (GO) terms of calcium (Ca^2+^) ion binding, cell redox homeostasis, and oxidoreductase activity especially for acting on NADPH were more significantly enriched (Figure 5B). Four genes from these GO terms (GO:0055114 and GO:0045454) were obtained, including *TraesCS4D02G324800*, *TraesCS1D02G2849 00*, *TraesCS5A02G301700*, and *TraesCS3D02G347900*, based on their putative biological properties in redox activity and salt response. These four genes encode NADPH oxidases, also known as Rbohs, putative homologs of *AtRbohD*, *AtRbohF*, and *AtRbohH* in Arabidopsis, which have been implicated in ROS production and signaling during plant abiotic stress response (Marino et al., 2012).

The significant enrichment of oxidoreductase activity genes that are affected by *TaHAG1* under salt stress suggests a pivotal role in ROS production and homeostasis in this condition. To test this hypothesis, we further compared the ROS content of *TaHAG1*-OE, *TaHAG1*-RNAi lines, and WT plants by histochemical stain. Both nitroblue tetrazolium (NBT) and 3,3′-diamino-bezidine (DAB) staining indicated three genotypes exhibit similar ROS contents under normal conditions; however, under salt stress conditions, both in the roots and leaves of *TaHAG1*-OE lines produce much higher amounts of hydrogen peroxide (H_2_O_2_) compared withWT, whereas *TaHAG1-*RNAi lines accumulate less H_2_O_2_ than WT plants (Figure 5, C and D). H_2_O_2_ production was also monitored using H_2_O_2_ content measurement and H_2_DCFDA labeling assay. These results are consistent with the histochemical staining in *TaHAG1*-OE, *TaHAG1*-RNAi lines, and WT plants before and after salt stress treatments (Figure 5E;Supplemental Figure S7), suggesting the production of ROS mediated by TaHAG1 is involved in wheat salt tolerance.

To further determine whether TaHAG1 confers wheat salt tolerance via ROS production, we tested the effect of H_2_O_2_ on salt tolerance in *TaHAG1*-OE, *TaHAG1*-RNAi lines, and WT plants (Figure 5F). NaCl treatment had a significant inhibition of roots growth in *TaHAG1*-RNAi lines than *TaHAG1*-OE and WT plants. However, H_2_O_2_ application together with NaCl treatment relieved the growth inhibition of three genotypes, especially ameliorated in *TaHAG1*-RNAi lines, exhibiting reduced leaf blade wilting and increased weight of root and shoot (Figure 5, F–I). Diphenylene iodonium (DPI) is a potent NADPH oxidase inhibitor, pretreatment with DPI prior to NaCl treatment aggravated the effect of salt damage on roots of three genotypes wheat, especially counteracted the salt tolerance advantages of *TaHAG1*-OE lines (Figure 5, F–I). The H_2_O_2_ application effect on *TaHAG1*-RNAi lines coupled with DPI treatment effect on *TaHAG1*-OE plants, further verifying the production of ROS mediated by TaHAG1 is responsible for wheat salt tolerance. Taken together, these results indicated TaHAG1 contributes to the salt tolerance of wheat by modulating the ROS production.

### Hexaploid wheat has increased ROS production than its tetraploid parent under salt stress

**Figure 6:**
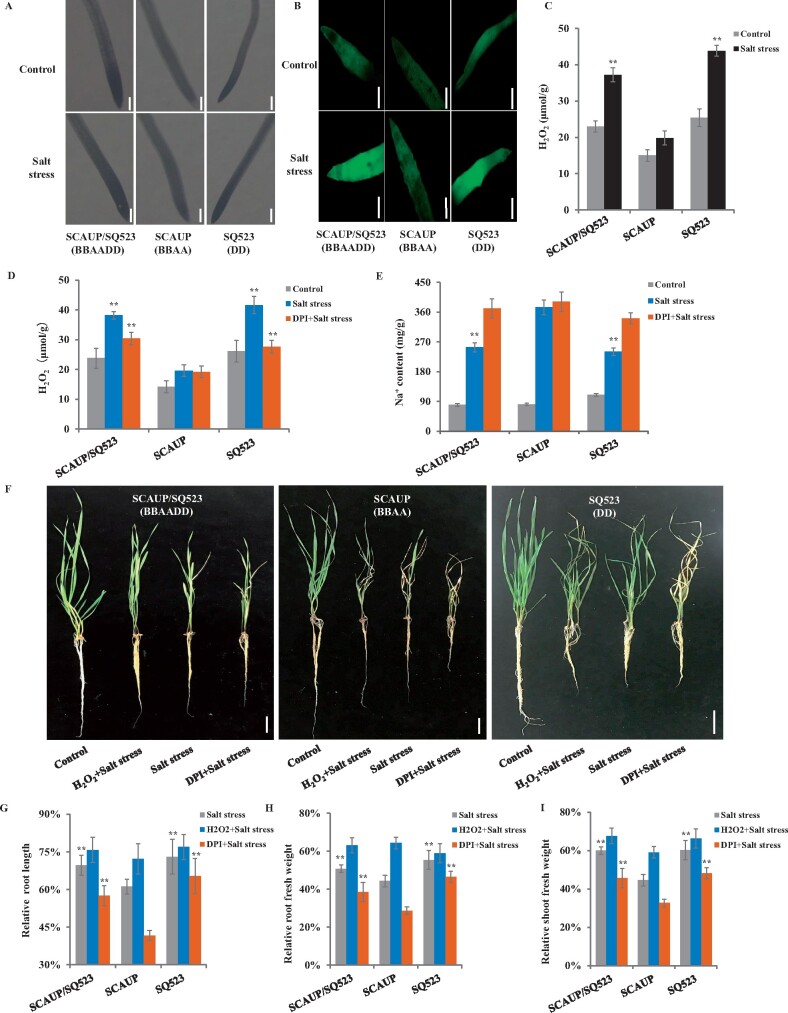
Synthetic hexaploid wheat with increased ROS production than its tetraploid parent under salt stress. A and B, NBT staining (A) and H2DCF-DA probe labeling (B) of NaCl-induced ROS production in roots of synthetic allohexaploid wheat SCAUP/SQ523, its tetraploid parent, *T. turgidum* SCAUP, and its diploid parent, *Ae. tauschii* SQ523 under control or NaCl treatment. Dark shading or fluorescence activity indicates the accumulation of H_2_O_2_. Scale bars, 500 μm. C, H_2_O_2_ contents in the seedlings of SCAUP/SQ523 with its parents SCAUP and SQ523 plants under control and salt stress conditions. In (A–C), 7-d-old seedlings grown in hydroponic culture were treated with 200 mM NaCl for 9 h. D and E, Effects of exogenous DPI on changes in H_2_O_2_ production (D) and Na^+^ accumulation (E) of synthetic allohexaploid wheat SCAUP/SQ523 and its parents SCAUP and SQ523. Seven-day-old seedlings grown in hydroponic culture were treated with 200 mM NaCl for 1 d or 10 μM DPI + 200 mM NaCl for 1 d, then the H_2_O_2_ contents and Na^+^ accumulation were quantitated. F, Effects of exogenous H_2_O_2_ and DPI on salt resistance of synthetic allohexaploid wheat SCAUP/SQ523 and its parents SCAUP and SQ523 plants. Nonstressed or stressed 7-d-old seedlings grown for 3 weeks and then photographed and quantitated. Scale bar, 5 cm. Salt stress: 200 mM NaCl; H_2_O_2_ + Salt stress: 10 mM H_2_O_2_ together with 200 mM NaCl; DPI + Salt stress: pre-treatment with 10 μM DPI prior to 200 mM NaCl treatment. G–I, Comparison of the seedlings growth of SCAUP/SQ523 with its parents SCAUP and SQ523 plants in corresponding treatment in (F). The *y*-axis denotes a percentage of stress-treated seedlings relative to the same genotype grown on control conditions. Root length (G), root fresh weight (H), and shoot fresh weight (I). Mean and sd values were derived from measurements of at least 15 individuals of each kind of genotype. Similar results were obtained from three independent biological experiments. Asterisks indicate significant differences between SCAUP/SQ523, SQ523 plants with SCAUP plants under the same treatment (*P < 0.05, **P < 0.01 by two-sided *t* test).

We further compared the ROS content between SCAUP/SQ523 with SCAUP and SQ523 under normal and salt conditions. Both histochemical staining and H2DCF-DA labeling assays indicated three genotypes exhibit similar ROS contents under normal conditions. However, under salt conditions, SCAUP/SQ523 and SQ523 show much higher amounts of H_2_O_2_ than SCAUP (Figure 6, A and B). The result was also verified by DAB staining (Supplemental Figure S8). In addition, under salt stress, the H_2_O_2_ amounts were increased within the three genotypes, but the H_2_O_2_ content was significantly higher in SCAUP/SQ523 and SQ523 than in SCAUP (Figure 6C), consistent with results of histochemical staining and H_2_O_2_ fluorescence activity.

### Inhibition of ROS production causes increased Na^+^ content and counteracts the salt tolerance advantages of hexaploid wheat

Previous studies indicated H_2_O_2_ is involved in the regulation of Na^+^ homeostasis under salt stress (Ma et al., 2012). We reasoned that the different levels of H_2_O_2_ between allohexaploid wheats and its tetraploid and diploid parents under salt stress may have an effect on their Na^+^ accumulation. To verify this hypothesis, DPI was applied on the SCAUP/SQ523 and SCAUP, SQ523 under salt stress. As shown in Figure 6D, the H_2_O_2_ content in SCAUP/SQ523 and SQ523 is significantly reduced after application of DPI under salt stress. Subsequently, the Na^+^ content increased significantly in SCAUP/SQ523 and SQ523, and close to that of SCAUP (Figure 6E). Together, these results suggest that H_2_O_2_ was required for the regulation of Na^+^ accumulation in polyploidy wheat under salt stress. Although the effect of DPI on H_2_O_2_ and Na^+^ accumulation in SCAUP under salt stress was negligible, consistent with its low capacity of H_2_O_2_ production under salt stress.

Furthermore, exogenous H_2_O_2_ treatment in salt stress conditions significantly relieved growth inhibition of three genotypes, especially ameliorated in SCAUP plants, including increased root and shoot weight, and relieved leaf blade wilting compared to NaCl treatment alone (Figure 6, F–I). In contrast, pretreatment with DPI prior to NaCl treatment aggravated the effect of salt damage on all tested genotypes, especially counteracted the salt tolerance advantages of SCAUP/SQ523 and SQ523 plants (Figure 6, F–I). These results indicated the difference in ROS content is part of the mechanism of the salt tolerance variation in wheat with varying ploidy.

### TaHAG1 is localized in nucleus and responsible for H3K9 and H3K14 acetylation in wheat

**Figure 7:**
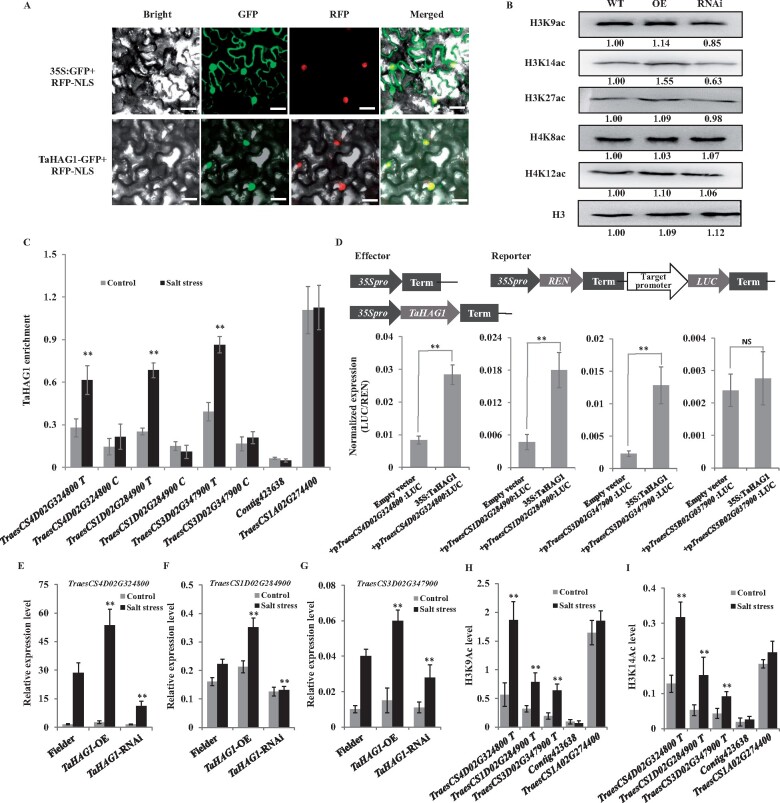
Cellular localization of TaHAG1 and the role of TaHAG1 in regulating target genes. A, Subcellular localization of TaHAG1. TaHAG1 (fused with GFP) was localized to the nucleus (colocalized with SPL14 fused with mCherry). Scale bars, 40 μm. B, Western blot analysis of histone acetylation levels in *TaHAG1*-OE, *TaHAG1*-RNAi lines, and WT Fielder plants with specific antibodies against histones H3K9ac, H3K14ac, and H3K27ac, H4K8ac, and H4K12ac were used. The levels of each histone modification were normalized to those of the WT. H3 was used as loading control. The numbers below the gel images indicate the relative abundance of the histone modification marks. C, Enrichment of TaHAG1 binding determined on the *TraesCS4D02G324800*, *TraesCS1D02G284900* and *TraesCS3D02G347900* loci by ChIP assays. Signals are given as percentages of the input chromatin value. T and C indicate TSSs and coding regions, respectively. *TraesCS1A02G274400* (*β-Actin*) and the *contig423638* (retrotransposon) are positive and negative control sites for TaHAG1 binding and histone acetylation enrichments, respectively (Hu et al., 2013). D, Dual LUC transcriptional activity assays to assess the ability of TaHAG1 to transactivate target gene expression. Schematic diagrams of the effector and reporter constructs. LUC/REN indicates the ratio of the signal detected for Firefly LUC and REN activity. Experiments were repeated at least three times for each reporter-effector combination (**P < 0.01 by Student’s *t* test). E–G, Transcript levels of *TraesCS4D02G324800*, *TraesCS1D02G284900*, and *TraesCS3D02G347900* genes in roots of WT Fielder, *TaHAG1*-OE, and *TaHAG1*-RNAi plants before and after salt stress treatments. Results are normalized with the *β-Actin*. Asterisks indicate significant differences between *TaHAG1* transgenic lines with WT plants under salt stress conditions (**P < 0.01 by two-sided *t* test). Samples were collected at the seedling stage. The values are means (±se) of three biological replicates. H and I, Enrichment of H3K9ac and H3K14ac on transcription initiation regions of the *TraesCS4D02G324800*, *TraesCS1D02G284900*, and *TraesCS3D02G347900* genes under salt stress conditions in WT Fielder plants. In (C), (H), and (I), Asterisks indicate significant differences between normal conditions and salt stress conditions (**P < 0.01 by Student’s *t* test); All error bars represent sd values drawn from at least three repetitions.

We carried out subcellular localization of TaHAG1 by fusing it to the GFP protein. The TaHAG1-GFP is colocalized to the nucleus with an RFP carrying a nuclear localization signal (Figure 7A), suggesting TaHAG1 functions in the nucleus. To determine whether TaHAG1 affects histone acetylation activity in wheat, the acetylation levels of H3K9, H3K14, H3K27 and H4K8, H4K12 were compared between the WT and the *TaHAG1* transgenic plants by western blots. The results showed an increase of H3K9 and H3K14 acetylation in the *TaHAG1*-OE lines, but decreased in *TaHAG1*-RNAi plants, compared with WT (Figure 7B). There is no significant difference in levels of H3K27, H4K8, and H4K12 acetylation between *TaHAG1*-OE, *TaHAG1*-RNAi lines, and WT plants (Figure 7B). These results suggest TaHAG1 is mainly involved in histone H3K9 and H3K14 acetylation in wheat.

### TaHAG1 directly targets three respiratory burst oxidase genes

To identify the direct target genes of TaHAG1 related to oxidation–reduction process under salt stress, 7-d-old seedlings of wheat treated with 0 or 200 mM NaCl for 2 d were collected for chromatin immunoprecipitation (ChIP) assay. The precipitated chromatin DNA was analyzed by quantitative PCR (qPCR) to examine enrichment levels relative to those of nonprecipitated (input) genomic DNA. The occupancy of TaHAG1 to the genomic regions near the transcriptional start sites (TSSs) and coding regions of four respiratory burst oxidase genes was analyzed (Supplemental Figure S9A). As shown in Figure 7C, relative high enrichment of the fragments near the TSSs of *TraesCS4D02G324800*, *TraesCS1D02G284900*, and *TraesCS3D02G347900* was detected in wheat Fielder under NaCl treatment compared with control conditions. In contrast, the enrichment was significantly declined in the coding regions of these genes before and after salt treatment (Figure 7C;Supplemental Figure S9A). These data suggest that TaHAG1 may specifically bind to the regions near the TSSs of these genes under salt stress. In addition, no significant enrichment was detected at the TSSs and coding region of *TraesCS5A02G301700* loci before and after salt treatment, suggesting it is not the direct target of TaHAG1 (Supplemental Figure S9B).

The effect of TaHAG1 regulating the target genes was also examined using a dual luciferase (LUC) transcriptional activity assay. The reporter plasmid harboring the promoter of target genes driving the expression of the LUC reporter gene was coexpressed with the effector *35S:TaHAG1* in *Nicotiana benthamiana* leaves. The LUC signal detection was used to determine transcriptional regulation. Transient LUC expression indicated that TaHAG1 can activate the expression of *TraesCS4D02G324800*, *TraesCS1D02G284900*, and *TraesCS3D02G347900* (Figure 7D). In contrast, TaHAG1 cannot activate *TraesCS5B02G037900* gene, since its expression was not affected by TaHAG1. Together, these results suggest that TaHAG1 is involved only in the expression of target genes in vivo.We further examined the expression of the *TraesCS4D02G324800*, *TraesCS1D02G284900*, and *TraesCS3D02G347900* in roots and leaves of *TaHAG1* transgenic lines and found they downregulated in *TaHAG1*-RNAi lines but upregulated in *TaHAG1*-OE lines compared to those in WT plants, especially under salt treatments (Figure 7, E–G; Supplemental Figure S9, C–E). As TaHAG1 is mainly involved in vivo in histone H3K9 and H3K14 acetylation in wheat, we further measured the H3 acetylation levels on transcription initiation regions of target genes by ChIP assays using the antibodies against H3K9ac and H3K14ac. The aforesaid genomic regions near the TSSs of *TraesCS4D02G324800*, *TraesCS1D02G284900*, and *TraesCS3D02G347900* genes were analyzed (Supplemental Figure S9A). As shown in Figure 7, H and I, the levels of H3K9ac and/or H3K14ac at TSSs of these genes were low in control conditions but were significantly increased under salt stress treatments. These changes indicated that the enrichment of TaHAG1 triggers increased H3K9ac, H3K14ac, and transcriptional upregulation of these loci. Collectively, based on these findings, we propose that TaHAG1 directly targets *TraesCS4D02G324800*, *TraesCS1D02G284900*, and *TraesCS3D02G347900* genes to trigger the epigenetic changes and in turn facilitates their expression in response to salt stress.

### Expressions of respiratory burst oxidase genes are positively associated with salt tolerance variation of wheat accessions with varying ploidy

According to the above results, we speculated that *Rboh* genes that regulated by TaHAG1 are involved in the modulation of salt resistance in wheat accessions with varying ploidy. We examined the expression of *TraesCS4D02G324800*, *TraesCS1D02G284900*, and *TraesCS3D02G347900* in SCAUP/SQ523 with its tetraploid and diploid parents. In normal conditions, these genes showed similar basal expression both in roots and leaves of three genotypes (Supplemental Figures S10, A–F and S11). However, under salt stress treatment, the transcripts both in roots and leaves were significantly higher in SCAUP/SQ523 and SQ523 than in SCAUP (Supplemental Figures S10, A–F and Figure S11), which are in line with the expression patterns of *TaHAG1*. In addition, we examined the expression changes of these genes in natural wheat accessions with varying ploidy before and after salt stress. Among these genes, the upregulation expression of *TraesCS4D02G324800* both in roots and leaves was significantly correlated with salt tolerance in wheat accessions with different ploidy (Figure S10, G–I). These results suggest the TaHAG1-regulated ROS production pathway is involved in salt tolerance difference of wheat accessions with varying ploidy.

### TaHAG1 maintains ROS homeostasis in wheat under salt stress

**Figure 8:**
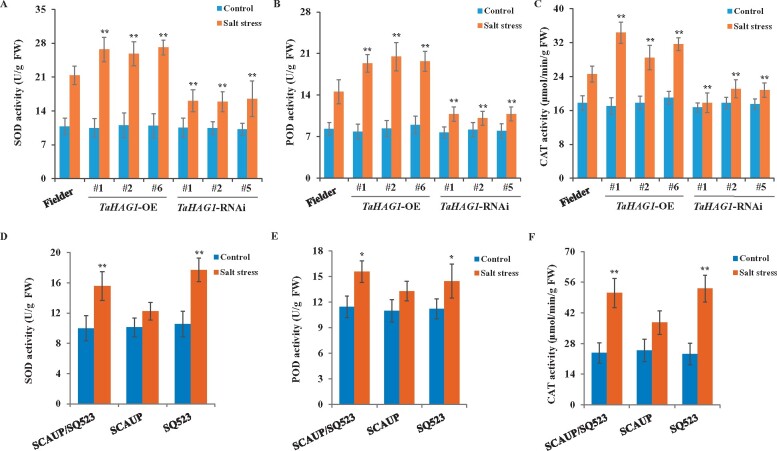
Hexaploid wheats and *TaHAG1*-OE lines with higher capacity of ROS homeostasis. A–C, Comparison of SOD (A), POD (B), and CAT (C) activity between *TaHAG1*-OE, *TaHAG1*-RNAi lines, and WT plants under normal and salt stress conditions. Asterisks indicate significant differences between *TaHAG1*-OE, *TaHAG1*-RNAi lines with WT plants under salt stress conditions. D–F, Comparison of SOD (D), POD (E), and CAT (F) activity of synthetic allohexaploid wheat SCAUP/SQ523, and its parents SCAUP, SQ523 plants under normal and salt stress conditions. Asterisks indicate significant differences between SCAUP/SQ523, SQ523 plants with SCAUP plants under salt stress conditions. Error bars indicate sd, *n* = 5. Statistical significance was determined by a two-sided *t* test: *P < 0.05, **P < 0.01.

Plant cell’s rapid accumulation of ROS in response to stress has an important role in inducing signaling events. However, the balance between ROS production and detoxification is critical for plant tolerance to stress. The cytosolic enzymatic antioxidants include super oxide dismutase (SOD), ascorbate peroxidase (POD), catalase (CAT) participate in ROS detoxification. Therefore, we further examined the activities of SOD, POD, and CAT in *TaHAG1*-OE, *TaHAG1*-RNAi, and WT plants. Under normal conditions, there were no significant differences between transgenic lines and WT plants (Figure 8, A–C). Under salt conditions, NaCl treatments clearly increased the activities of these antioxidants in all these plants, especially for SOD and POD. Notably, the increased magnitude of SOD, POD, and CAT activities in *TaHAG1*-OE lines was remarkably higher than that in the WT plants, while *TaHAG1*-RNAi lines had lower activities of SOD, POD, and CAT than WT (Figure 8, A–C). These results suggest that the *TaHAG1*-OE lines are more effective than WT and *TaHAG1*-RNAi lines in terms of ROS detoxification.

### Hexaploid wheats with higher capacity of ROS scavenging than that in tetraploids

To address the possibility that ROS were detoxified more effectively in hexaploid wheat than in tetraploids, we also measured the activities of SOD, POD, and CAT in synthetic allohexaploid wheat with their parents both in normal and salt stress conditions. There were no significant differences between SCAUP/SQ523 and SCAUP, SQ523 in normal conditions (Figure 8, D–F). However, under salt stress, the antioxidant enzyme activities were enhanced in all three genotypes, but SCAUP/SQ523 and SQ523 showed higher SOD, POD, and CAT activities than those in SCAUP (Figure 8, D–F). These results suggest that under salt conditions the hexaploid wheat and diploid parent with greater detoxification ability than the tetraploid wheat.

## Discussion

It is generally believed that polyploidy species has the superiority for the adaptation to a wider range of environmental conditions, thus providing the potential for evolutionary advantages (Dubcovsky and Dvorak, 2007; Feldman et al., 2012; Yang et al., 2018). This has clearly been the case for hexaploid bread wheat, which combines the D genome from *Ae. tauschii* with the AB genomes from tetraploid wheat. Compared with tetraploid wheat, hexaploid bread wheat has broader adaptability to different environments and especially exhibits enhanced fitness and more salt-tolerant phenotypes (Munns et al., 2012; Yang et al., 2014). The molecular basis underlying this trait has always been a topic of great interest to scientists. Many attempts have been made to identify the genes associated with salt tolerance and controlling these processes. Previous studies indicated bread wheat accumulates less Na^+^ and more K^+^ in leaves than durum wheat, and higher K^+^/Na^+^ ratios in leaves of bread wheat correspond with its higher salt tolerance (Dvorak et al., 1994). Further analysis indicated this trait was governed by *Kna1* locus on chromosome 4DL (Dubcovsky et al., 1996). Genetic and comparative mapping analysis also indicates that the D genome makes a major contribution to the strengthened salt tolerance in allohexaploid wheat, and speculate the improved K^+^/Na^+^ ratios of hexaploid wheat can be attributed specifically to the D genome, then this character appears to be dominant in the hexaploid wheats, where it is in competition with the A and B genomes (Shan et al., 1987; Dubcovsky et al., 1996; Huang et al., 2008; Munns et al., 2012; Byrt et al., 2014; Yang et al., 2014). Our results are consistent with the previous study, which showed that hexaploid wheats resembled more closely the diploid species *Ae. tauschii* than the allotetraploid wheat under salt stress treatment, suggesting that the strengthened salt tolerance of hexaploid wheat is mainly inherited from D genome donor. Further studies indicated a large proportion of the well-characterized mechanisms that confer enhanced salt tolerance of plant belong to the HKT-type transporters (James et al., 2006; Huang et al., 2008; Byrt et al., 2014), *TaHKT1;5* and *TaHKT1;4* are two relatively well-studied Na^+^-selective transporters that involved in the essential salinity tolerance in wheat. In this study, our results also show that both the natural and synthetic hexaploid wheat exhibit greater salinity tolerance than their tetraploid progenitors. We report the identification of a histone acetyltransferase TaHAG1 and its roles in the regulation of polyploidy wheat salt tolerance. However, our study indicates TaHAG1 did not regulate salt tolerance by regulating *TaHKT1;5* or *TaHKT1;4*, because there was no distinct change in expression of *TaHKT1;5* or *TaHKT1;4* between the WT and *TaHAG1*-OE or *TaHAG1*-RNAi under salt stress (Supplemental Figure S12), suggesting TaHAG1 contributes to wheat salt tolerance through a different pathway.

### TaHAG1 contributes to salt tolerance of hexaploid wheat via ROS production

Increasing evidences suggest that ROS are involved in the regulation of K^+^/Na^+^ balance and contribution for salt tolerance. For instance, the accumulation of H_2_O_2_ is required for retaining high K^+^/Na^+^ homeostasis and increased salt resistance in *Populus euphratica* (Zhang et al., 2007). reported bread wheat introgression line SR3 exhibits better fitness under salt conditions than variety JN177 by modulating ROS content. Recently, a study in cucumber reveals that treatment with H_2_O_2_ (10 mM) markedly enhanced salt tolerance (Zhang et al., 2020). These data indicate that plants treated with the appropriate concentration of H_2_O_2_ could significantly attenuate salt-induced damage, suggesting H_2_O_2_ can act as a molecular signal to trigger regulatory pathway in response to salt stress. Furthermore, it has been uncovered the existence of an H_2_O_2_-dependent long-distance signal induced by various abiotic stimuli, the accumulation of H_2_O_2_ in the extracellular spaces generates a “ROS wave.” The ROS wave functions as a general priming signal in plants, alerting systemic tissues to the occurrence of a localized abiotic stress stimulus (Mittler et al., 2011; Baxter et al., 2014; Waszczak et al., 2018). Despite the wealth of information on the vital role of ROS in responses to abiotic stress, little is known about its role in wider adaptability of polyploidy to diverse environments. In this study, we revealed H_2_O_2_ content is positively associated with variation of salt tolerance in polyploid wheat and described the role of TaHAG1 in the regulation of polyploidy wheat salt tolerance by modulating the ROS production and homeostasis. Our results showed that knock-down of *TaHAG1* decreases H_2_O_2_ production and diminishes K^+^/Na^+^ ratio, while overexpression of *TaHAG1* results in H_2_O_2_ accumulation and improved tolerance to salt stress (Figure 5). Notably, the synthetic hexaploid wheat SCAUP/SQ523 and diploid SQ523 with higher *TaHAG1* expression also show much higher levels of H_2_O_2_ than tetraploid SCAUP under salt stress condition (Figure 6). Furthermore, exogenous H_2_O_2_ treatment could attenuate salt-induced damage to *TaHAG1*-RNAi lines and tetraploid SCAUP plants. In contrast, application of NADPH oxidase inhibitor DPI clearly reduced H_2_O_2_ contents, then increased Na^+^ contents and counteracted the salt tolerance advantages of *TaHAG1*-OE lines and SCAUP/SQ523 plants. Taken together, these findings demonstrate that ROS production medicated by TaHAG1 is a positive signal molecule to modulate Na^+^ homeostasis in polyploidy wheats under salinity conditions.

The *Rboh* genes or NADPH oxidases are considered to be responsible for most ROS production (Foreman et al., 2003; Sagi and Fluhr, 2006). In Arabidopsis, NADPH oxidase AtRbohD and AtRbohF function in ROS-dependent modulation of salt tolerance through maintaining K^+^/Na^+^ homeostasis (Ma et al., 2012). AtrbohC also has been shown to regulate salt tolerance by contributing to the mRNA stability of Na^+^/H^+^ anti-transporter *SOS1* (Chung et al., 2008). Here, we demonstrate that three NADPH oxidase genes are direct targets of TaHAG1 and that it plays a critical role in salt tolerance of wheat. Knock-down of *TaHAG1* considerably suppresses these genes expression, which was responsible for ROS production. ChIP assays indicate these *Rboh* genes are direct targets that are regulated by TaHAG1. Furthermore, enrichment of TaHAG1 triggers increased H3K9ac, H3K14ac, and transcriptional upregulation of these loci under salt treatments, which provides further epigenetic regulatory evidence that TaHAG1 contributes to ROS production and salt tolerance in wheat. Our work shows an example of how epigenetic remodeling factor is involved in the adaptability advantages of hexaploid wheat. We also found that several genes that are involved in Ca^2+^ ion binding and Ca^2+^ influx signaling pathways, including calmodulin (*TraesCS2B02G192200*), Ca^2+^-dependent protein kinase (*TraesCS6D02G152100*), and calcium-binding EF-hand protein (*TraesCS2B02G458100*), were significantly upregulated in *TaHAG1*-OE plants under salt treatments (Supplemental Table S4), implying that these genes and involved pathways might also participate in the TaHAG1-mediated salt stress response, an area that deserves further study.

Excessive ROS could cause oxidative damage to the tissues, Thus, the balance between ROS production and detoxification is critical for plant tolerance to stress. Intriguingly, TaHAG1 achieves the ROS homeostasis under salt stress likely by promoting the activities of cytosolic enzymatic antioxidants, including SOD, POD, and CAT, although it is still unclear whether TaHAG1 directly regulates the crucial genes that encoded cytosolic enzymatic antioxidants or involved in ROS scavenge pathways. Our study indicated the higher expression of *TaHAG1* in overexpression lines and synthetic hexaploid wheat confer their efficient antioxidant capability and ensure ROS homeostasis under salt conditions, and this characteristic also verified by the lower MDA content in synthetic hexaploid wheats than their tetraploid parents under salt conditions (Figures 1 and 8).

### Application of the *TaHAG1*-mediated salt tolerance in wheat

Understanding how homeologs are differentially expressed in response to ambient conditions and how artificial selection affects epigenetic changes in polyploid crops will facilitate plant breeding and crop improvement. Studies increasingly suggest that differential expression of the homeologs that due to inherent parental expression divergence, reorganized interactions of parental cis- and trans-regulatory factors, or de novo epigenetic remodeling, may affect the fitness of the newly formed allopolyploid and increase its competitiveness, leading to its successful establishment in nature (Birchler, 2012; Feldman et al., 2012; Hu et al., 2013; Han et al., 2016; Powell et al., 2017; Uauy, 2017; Ding and Chen, 2018; Yang et al., 2018). This implicating the reorchestration of homeologous gene expression plays an important role in the evolutionary advantage of hexaploid wheat. Our results indicated the transcript of *TaHAG1* was significantly higher in hexaploid wheat and diploid *Ae. tauschii* than tetraploid wheat after salt stress, implying the expression of *TaHAG1-A*, *TaHAG1-B*, and *TaHAG1-D* may exhibit divergence under salt treatment, and *TaHAG1-D* could response significantly than *TaHAG1-A* and *TaHAG1-B* in salt treatment. The three homeologs of *TaHAG1* share high coding sequence similarity, but distinct in promoters and there are several putative cis-acting elements that were detected only in *TaHAG1-D* homeolog (Supplemental Figure S13). Further investigations are required to dissect the possible upstream regulators that act differently on the promoters of *TaHAG1* homeologs under salt stress.

Enhancement of salt tolerance by modification of target genes often comes with compromise in plant growth and yield. For instance, mutations in rice *OsRR9* and *OsRR10* enhanced salinity tolerance but resulted in reduced panicle number and spikelet number per panicle (Wang et al., 2019). Our results showed that expression of *TaHAG1* confers a yield advantage under salt stress conditions, without observable penalty on plant growth and development. This result suggests *TaHAG1* and its potential favorable homeolog *TaHAG1-D* can serve as a direct target for both genetic engineering and selection for improvement of wheat salt tolerance. The regulatory factors or genes involved in salt tolerance identified in this study may also be of great value for genetic improvement in wheat as well as in other crops.

## Materials and methods

### Plant materials and growth conditions

The synthetic allohexaploid wheat (*T. aestivum*) SCAUP/SQ523 and its tetraploid SCAUP and diploid SQ523 progenitors, 48 natural wheat accessions with different ploidy were used to examine the variation of salt tolerance. The surface-sterilized seeds were incubated at 4°C for 3 d in dark and then exposed to white light at room temperature. Germinated seeds were transplanted into pots and grown in greenhouse with a 12-h photoperiod of cool white fluorescent and incandescent light exposure (intensity >3,000 lx) and 60% humidity.

### Salt treatment

For phenotypic analysis in seedling stage, the seeds of different accessions were planted in pots filled with uniformly mixed Pindstrup substrate (nine plants for each) and grown with a 12 h photoperiod at 20°C in greenhouse. The 7-d-old uniform seedlings were treated with 0 or 200-mM NaCl solution for 3 weeks. Then the shoot weight, root length, and weight were quantified and photos were taken.

For phenotypic analysis in vegetative and reproductive stages, the salt tolerance of WT and different transgenic lines were phenotyped in three independent repeats. Each independent repeat contained eight seedlings and was sowed in plastic pots filled with uniformly mixed Pindstrup substrate. The 10-d-old seedlings were supplied with 250 mM NaCl solution every 4 d and plants were continuously subjected to salt stress from seedling to maturity. In control conditions, watering was continued every 4 d throughout the experiment. All pots were placed in greenhouse under a 12-h light cycle at 20°C. The plants were photographed, and the fitness phenotypes of 15 individuals of each genotype after ripening were assessed, including the plant height, spike length, and grain weight per spike.

### Measurements of MDA, electrolyte leakage, ion, and chlorophyll content

MDA contents were determined using thiobarbituric acid as previously described (Wang et al., 2017). Chlorophyll was measured by aqueous acetone (80%, m/v) as described earlier (Arnon, 1949). Electrolyte leakage was measured according to the previously described methods (Nishiyama et al., 2011). The shoot and root of samples were harvested, then dissolved in HNO_3_ and analyzed by inductively coupled plasma optical emission spectrometry (FP6410, INESA).

### Genomic DNA and total RNA extraction

The genomic DNA of different wheat genotypes was extracted using the CTAB protocol. Total RNA was extracted with TRIzol reagent (Invitrogen, Carlsbad, CA), and purified RNA was treated with DNase I. Subsequently, first-strand cDNA synthesis was performed using HiScipt II One-Step RT-PCR Kit (Vazyme Biotech, Nanjing, China).

### Reverse transcription qPCR

The samples were collected after treated with 200 mM NaCl at indicated time point. Total RNA was extracted with TRIzol (Invitrogen), and purified RNA was treated with DNase I. First-strand cDNA synthesis was performed using HiScipt II One-Step RT-PCR Kit (Vazyme Biotech). Reverse transcription qPCR (RT-qPCR) analysis was conducted using SYBR Premix EX TagTM (TaKaRa, Kyoto, Japan) in a volume of 20 μL using Bio-Rad CFX96 system. Gene primers for qPCR were designed according to the conserved region of three homeologs. Relevant primer sequences were given in Supplemental Table S5. Expression levels were normalized to those of *Actin* and *tubulin*, respectively. Each sample was quantified at least in triplicate.

### Plasmid construction and plant transformation

The ORF of *TaHAG1-D* was amplified and inserted into the pMWB122 vector using *BamH* I and *Sac* I sites to achieve the *Ubi:TaHAG1* construct. For *TaHAG1-*RNAi, the inverted repeat DNA construct for *TaHAG1* was ligated into the intermediate vector pMWB006, then digested with *Hind* III and cloned into pMWB111 vector. The CRISPR-Cas9 knockout lines were created by Prof. Yingyin Yao in China Agricultural University. In essence, reverse complementary sgRNA sequences with *Bsa* I cohesive ends were synthesized, then oligonucleotides were annealed and inserted into the terminal vector pBUE411. All binary vectors harboring the desired constructs were transferred into strain EHA105 and transformed into the wheat cultivar Fielder using *Agrobacterium*-mediated transformation.

### RNA-seq and statistical analysis

Seven-day-old seedlings of the bread wheat Fielder and *TaHAG1*-OE lines were treated with 200 mM NaCl solution, the seedlings were collected 0 and 2 d after treatments for RNA extraction. Two biological replicates were performed for each sample. At least 2-µg total RNA of each replication was constructed cDNA libraries by using Illumina Poly-A Purification TruSeq library reagents, and performed on NovaSeq platform. Raw reads obtained from Illumina sequencing were processed and filtered using Trimmomatic with “SLIDINGWINDOW:4:20 MINLEN:40” to generate high-quality reads. The RNA-seq reads were aligned to the Chinese Spring reference genome (International Wheat Genome Sequencing Consortium RefSeq version 1.1) using STAR with default parameters. The read counts were normalized to FPKM values to show the relative gene expression levels and detailed analysis of RNA-seq data as previously described (Robinson et al., 2010). The differentially expressed genes between *TaHAG1*-OE line and WT were characterized by the Bioconductor package “edgeR” and agriGO version 2.0 (cutoff of FDR < 0.01) were used to carried out GO analysis. Differential expression of RPKM normalized data was tested by ANOVA analysis. The statistically significant difference was set at the *P* < 0.01 confidence level.

### Histochemical detection of ROS

The seedlings were treated with 200 mM NaCl for 3 h (for root stain) or 24 h (for leaf stain) in 1/2 Hoagland solution before staining. For DAB staining, the samples were covered with 1 mg/mL DAB for 18 h in the dark at 22°C, and then in 95% ethanol to decolor. For NBT staining, the samples were first immersed in NBT stain solution until the dark blue color appeared, and then in 95% ethanol to decolor.

### H_2_O_2_ content quantification and monitored by fluorescence probe

H_2_O_2_ content was determined following the protocol of the H_2_O_2_ Colorimetric Assay Kit (COMIN, Hallettsville, TX). The H_2_O_2_-specific fluorescent probe, H_2_DCF-DA (2′,7′-dichlorodihydro fluorescein diacetate), was used to detect H_2_O_2_ accumulation in plants. Seven-day-old seedlings were exposed to 200 mM NaCl for 3 h. Seedlings were stained with 10 µM H_2_DCF-DA in the dark for 10 min and then washed three times with HBSS solution. The fluorescence was examined by epifluorescence microscopy.

### H_2_O_2_ and DPI treatments

For phenotype observation and physiological parameters measurement, 7-d-old seedlings were divided into four groups. Group I as control watered with 1/2 Hoagland solution. Group II was treated with 200 mM NaCl. Group III was treated with 200 mM NaCl and sprayed H_2_O_2_ (10 mM) on the leaf surface every other day. Group IV was pretreated with 10 µM DPI for 6 h, then supplemented with 200 mM NaCl. Na^+^ and H_2_O_2_ contents were measured after 9 h. Morphological and physiological parameters were measured and samples were collected after 3 weeks.

### Subcellular localization

The cDNA of *TaHAG1* was cloned into between *EcoR* I and *Hind* III sites of the super1300 vector to generate p*35S:TaHAG1-GFP*. The fusion construct was co-transformed into *N. benthamiana* leaves. For controls, leaves were transformed with p35S:SPL14-mCherry (RFP) and super1300 (p*35S:GFP*). Fluorescence was examined at the wavelength of 480–520 and 540–600 nm to detect GFP and RFP signal, respectively, using confocal microscopy (LSM 510 META; Zeiss, Oberkochen, Germany) at 16 h after transformation.

### Western blot analysis

The seedling of WT Fielder, *TaHAG1*-OE, and *TaHAG1*-RNAi lines was used to extract total protein. Harvested samples were ground in liquid nitrogen and proteins were extracted with lysis buffer (50 mM of Tris–HCl at pH 6.8, 10% [v/v] glycerol, 2% [w/v] SDS, and 5% [v/v] β-mercaptoethanol) at 100°C for 5 min. The protein separation, polyvinylidene fluoride membrane transferring, and incubation detailed steps as previously described (Liu et al., 2019). After washing with 1×TBST, the secondary antibody (1:3,000) was added for 1 h at room temperature, followed by 1×TBST washing. Two-component reagent-clarity western ECL Substrate (Cat. no.170-5060; Bio-Rad, Hercules, CA, USA) was used for detection. The signal was detected with X-ray film.

### ChIP assays

For each assay, fresh seedling materials were collected and subjected to vacuum infiltration in 1% (v/v) formaldehyde for 15 min to crosslink the chromatin proteins to DNA. Chromatin was extracted and sonicated using Branson Sonifier. The average size of the resultant DNA fragments was sheared into 0.2–1.0 kb. The detailed ChIP assays were accomplished as previously described (Hu et al., 2013). The antibody to TaHAG1 was custom-made by immunizing rabbits in Wuxi Pharma Tech Company (Shanghai, China) and specificity of monoclonal antibodies was tested as previously described (Hu et al., 2015). The anti-H3K9ac and H3K14ac were purchased from Upstate Biotechnology (Lake Placid, NY, USA). Immunoprecipitated DNA was analyzed by qPCR and amplified DNA from the chromatin fractions prior to antibody incubation were used as the controls. PCR reactions were performed in triplicate for each sample, and the enrichments were normalized to the input sample.

### Transient expression assays

Dual LUC reporter assays were performed as previously described (Liu et al., 2019). Approximately 2-kb fragments upstream of the TSS in target genes were amplified and cloned into pGreenII 0800-LUC, generating the reporter constructs, respectively. The *TaHAG1* ORF was cloned into pUC18-35S vector generating *35S:TaHAG1* effector construct. Four-week-old *N. benthamiana* plants were coinfiltrated with GV3101 harboring the desired reporter and effector or empty vector. The Firefly and Renilla LUC (REN) were quantified using Dual-LUC Reporter Assay system (Promega, Madison, WI, USA). Reporter LUC activities were standardized by activities of co-expressed REN LUC.

### Enzyme activity assays

The antioxidants enzyme activity, including SOD, POD, and CAT were measured using the POD Activity Assay Kit, CAT Activity Assay Kit, Superoxide Dismutase Activity Assay Kit (COMIN), respectively. Samples were prepared according to the manufacturer’s instructions. Total POD activity was measured by monitoring the oxidation of 3,30-dimethoxybenzidine at 470 nm. Total CAT activity was assayed by measuring the rate of decomposition of H_2_O_2_ at 240 nm. SOD activity was determined by measuring the percentage of inhibition of the pyrogallol autoxidation.

## Accession numbers

Sequence data from this article can be found in the Ensembl data libraries under accession numbers *TaHAG1* (TraesCS1D02G134200), *TaRbohD* (TraesCS4D02G324800), *TaRbohF* (TraesCS1D02G284900), and *TaRbohH* (TraesCS3D02G347900).

## Supplemental data

The following supplemental materials are available in the online version of this article.

**Supplemental Figure S1**. Comparative analyses of the relative shoot fresh weight for natural wheat accessions with varying ploidy under salt stress.

**Supplemental Figure S2**. Venn diagram showing overlap of upregulated genes between SCAUP/SQ523 with its allotetraploid parent SCAUP or with its diploid parent SQ523 in response to salt stress.

**Supplemental Figure S3**. Sequence analysis of *TaHAG1* homeologs.

**Supplemental Figure S4**. Expression pattern of *TaHAG1* in wheat accessions with different ploidy under salt stress.

**Supplemental Figure S5**. Identification of the role of *TaHAG1* in wheat salt tolerance.

**Supplemental Figure S6**. Knockout of *TaHAG1* causes reduced salt tolerance in wheat.

**Supplemental Figure S7**. ROS production detected by H2DCF-DA fluorescence in roots of the WT Fielder and *TaHAG1*-OE, *TaHAG1*-RNAi plants under control or salt stress conditions.

**Supplemental Figure S8**. DAB staining of NaCl-induced ROS production in roots of SCAUP/SQ523, SCAUP, and SQ523 under salt stress conditions.

**Supplemental Figure S9**. TaHAG1 enrichment and expression pattern analysis of the target genes.

**Supplemental Figure S10**. Salinity-induced respiratory burst oxidase gene expression was correlated with salt tolerance variation in polyploidy wheat.

**Supplemental Figure S11**. Transcript levels of three TaHAG1 target genes in synthetic allopolyploid wheat SCAUP/SQ523 and their parents SCAUP, SQ523 under salt stress treatment.

**Supplemental Figure S12**. Expression pattern of *TaHKT1;5* and *TaHKT1;4* in *TaHAG1* transgenic lines under salt stress.

**Supplemental Figure S13**. The cis-acting regulatory elements analysis for promoter sequences of *TaHAG1-A*, *TaHAG1-B*, and *TaHAG1-D* genes from hexaploid wheat genotype Fielder using PlantCARE database.

**Supplemental Table S1**. The information of wheat accessions with varying ploidy used for salt tolerance analysis.

**Supplemental Table S2**. List of upregulated genes response to salt stress in SCAUP/SQ523, SCAUP, and SQ523.

**Supplemental Table S3**. List of differentially expressed genes response to salt stress in WT Fielder and *TaHAG1*-OE lines plants.

**Supplemental Table S4**. List of TaHAG1-regulated genes in response to salt stress.

**Supplemental Table S5**. Gene-specific primer pairs used in this study.

## Supplementary Material

kiab187_Supplementary_Data

## Notes

M.Z., J.C.L., X.B.L., W.C., J.P.L., and W.J.S. performed the experiments; Y.J.G., K.X.A., M.M.X., Y.Y.Y., H.R.P., and Z.F.N. contributed to materials; Z.R.H., M.Z., and Q.X.S. conceived the research and analyzed the data; Z.R.H. wrote the paper with input from Q.X.S.

The author responsible for distribution of materials integral to the findings presented in this article in accordance with the policy described in the Instructions for Authors (https://academic.oup.com/plphys/pages/general-instructions) is: Zhaorong Hu (zrhu@cau.edu.cn).

## References

[kiab187-B1] Adams KL , Wendel JF ( 2005) Polyploidy and genome evolution in plants. Curr Opin Plant Biol 8:135–141 15752992 10.1016/j.pbi.2005.01.001

[kiab187-B2] Apel K , Hirt H ( 2004) Reactive oxygen species: metabolism, oxidative stress, and signal transduction. Annu Rev Plant Biol 55: 373–399 15377225 10.1146/annurev.arplant.55.031903.141701

[kiab187-B3] Arnon D ( 1949) Copper enzymes in isolated chloroplasts. Polyphenoloxidase in Beta vulgaris. Plant Physiol 24: 1–15 16654194 10.1104/pp.24.1.1 PMC437905

[kiab187-B4] Baxter A , Mittler R, Suzuki N ( 2014) ROS as key players in plant stress signalling. J Exp Bot 65: 1229–1240 24253197 10.1093/jxb/ert375

[kiab187-B5] Berkman PJ , Visendi P, Lee HC, Stiller J, Manoli S, Lorenc MT, Lai K, Batley J, Fleury D, Simková H et al (2013) Dispersion and domestication shaped the genome of bread wheat. Plant Biotechnol J 11: 564–571 23346876 10.1111/pbi.12044

[kiab187-B6] Birchler JA ( 2012) Insights from paleogenomic and population studies into the consequences of dosage sensitive gene expression in plants. Curr Opin Plant Biol 15: 544–548 22939251 10.1016/j.pbi.2012.08.005

[kiab187-B7] Byrt CS , Xu B, Krishnan M, Lightfoot DJ, Athman A, Jacobs AK, Watson-Haigh NS, Plett D, Munns R, Tester M et al (2014) The Na^+^ transporter, TaHKT1;5-D, limits shoot Na^+^ accumulation in bread wheat. Plant J 80: 516–526 25158883 10.1111/tpj.12651

[kiab187-B8] Chalhoub B , Denoeud F, Liu S, Parkin IA, Tang H, Wang X, Chiquet J, Belcram H, Tong C, Samans B, et al (2014) Early allopolyploid evolution in the post-Neolithic *Brassica napus* oilseed genome. Science 345: 950–953 25146293 10.1126/science.1253435

[kiab187-B9] Chang YN , Zhu C, Jiang J, Zhang H, Zhu JK, Duan CG ( 2020). Epigenetic regulation in plant abiotic stress responses. J Integr Plant Biol 62: 563–580 31872527 10.1111/jipb.12901

[kiab187-B10] Chao DY , Dilkes B, Luo H, Douglas A, Yakubova E, Lahner B, Salt DE ( 2013) Polyploids exhibit higher potassium uptake and salinity tolerance in Arabidopsis. Science 341: 658–659 23887874 10.1126/science.1240561 PMC4018534

[kiab187-B11] Cheng X , Zhang S, Tao W, Zhang X, Liu J, Sun J, Zhang H, Pu L, Huang R, Chen T ( 2018) INDETERMINATE SPIKELET1 recruits histone deacetylase and a transcriptional repression complex to regulate rice salt tolerance. Plant Physiol 178: 824–837 30061119 10.1104/pp.18.00324 PMC6181036

[kiab187-B12] Chung JS , Zhu JK, Bressan RA, Hasegawa PM, Shi H ( 2008) Reactive oxygen species mediate Na^+^-induced SOS1 mRNA stability in Arabidopsis. Plant J 53: 554–565 17996020 10.1111/j.1365-313X.2007.03364.x PMC3128381

[kiab187-B13] Ding M , Chen ZJ ( 2018) Epigenetic perspectives on the evolution and domestication of polyploid plant and crops. Curr Opin Plant Biol 42: 37–48 29502038 10.1016/j.pbi.2018.02.003 PMC6058195

[kiab187-B14] Dubcovsky J , Dvorak J ( 2007) Genome plasticity a key factor in the success of polyploid wheat under domestication. Science 316: 1862–1866 17600208 10.1126/science.1143986 PMC4737438

[kiab187-B15] Dubcovsky J , María GS, Epstein E, Luo MC, Dvořák J ( 1996) Mapping of the K^+^/Na^+^ discrimination locus *Kna1* in wheat. Theor Appl Genet 92: 448–454 24166270 10.1007/BF00223692

[kiab187-B16] Dvorak J , Noaman MM, Goyal S, Gorham J ( 1994) Enhancement of the salt tolerance of *Triticum turgidum* L. by the *Kna1* locus transferred from the *Triticum aestivum* L. chromosome 4D by homoeologous recombination. Theor Appl Genet 87: 872–877 24190475 10.1007/BF00221141

[kiab187-B17] Feldman M , Levy AA, Fahima T, Korol A ( 2012) Genomic asymmetry in allopolyploid plants: wheat as a model. J Exp Bot 63: 5045–5059 22859676 10.1093/jxb/ers192

[kiab187-B18] Foreman J , Demidchik V, Bothwell JH, Mylona P, Miedema H, Torres MA, Linstead P, Costa S, Brownlee C, Jones JD et al (2003) Reactive oxygen species produced by NADPH oxidase regulate plant cell growth. Nature 422: 442–446 12660786 10.1038/nature01485

[kiab187-B19] Guo W , Xin M, Wang Z, Yao Y, Hu Z, Song W, Yu K, Chen Y, Wang X, Guan P et al (2020) Origin and adaptation to high altitude of Tibetan semi-wild wheat. Nat Commun 11: 5085 33033250 10.1038/s41467-020-18738-5 PMC7545183

[kiab187-B20] Han Y , Xin M, Huang K, Xu Y, Liu Z, Hu Z, Yao Y, Peng H, Ni Z, Sun Q ( 2016) Altered expression of *TaRSL4* gene by genome interplay shapes root hair length in allopolyploid wheat. New Phytol 209: 721–732 26334764 10.1111/nph.13615

[kiab187-B21] Hou H , Zheng X, Zhang H, Yue M, Hu Y, Zhou H, Wang Q, Xie C, Wang P, Li L ( 2017) Histone Deacetylase is required for GA-induced programmed cell death in maize aleurone layers. Plant Physiol 175: 1484–1496 28972079 10.1104/pp.17.00953 PMC5664472

[kiab187-B22] Hu Z , Han Z, Song N, Chai L, Yao Y, Peng H, Ni Z, Sun Q ( 2013) Epigenetic modification contributes to the expression divergence of three TaEXPA1 homoeologs in hexaploid wheat (*Triticum aestivum*). New Phytol 197: 1344–1352 23360546 10.1111/nph.12131

[kiab187-B23] Hu Z , Song N, Zheng M, Liu X, Liu Z, Xing J, Ma J, Guo W, Yao Y, Peng H, Xin M, Zhou DX, Ni Z, Sun Q ( 2015) Histone acetyltransferase GCN5 is essential for heat stress-responsive gene activation and thermotolerance in Arabidopsis. Plant J 84: 1178–1191 26576681 10.1111/tpj.13076

[kiab187-B24] Huang S , Spielmeyer W, Lagudah ES, Munns R ( 2008) Comparative mapping of HKT genes in wheat, barley and rice, key determinants of Na^+^ transport and salt tolerance. J Exp Bot 59: 927–937 18325922 10.1093/jxb/ern033

[kiab187-B25] James RA , Davenport RJ, Munns R ( 2006) Physiological characterization of two genes for Na^+^ exclusion in durum wheat, *Nax1* and *Nax2*. Plant Physiol 142: 1537–1547 17028150 10.1104/pp.106.086538 PMC1676036

[kiab187-B26] Jiao W , Yuan J, Jiang S, Liu Y, Wang L, Liu M, Zheng D, Ye W, Wang X, Chen ZJ ( 2018) Asymmetrical changes of gene expression, small RNAs and chromatin in two resynthesized wheat allotetraploids. Plant J 93: 828–842 29265531 10.1111/tpj.13805

[kiab187-B27] Jiao Y , Wickett NJ, Ayyampalayam S, Chanderbali AS, Landherr L, Ralph PE, Tomsho LP, Hu Y, Liang H, Soltis PS et al (2011). Ancestral polyploidy in seed plants and angiosperms. Nature 473: 97–100 21478875 10.1038/nature09916

[kiab187-B28] Kim JY , Yang W, Forner J, Lohmann JU, Noh B, Noh YS ( 2018) Epigenetic reprogramming by histone acetyltransferase HAG1/AtGCN5 is required for pluripotency acquisition in Arabidopsis. EMBO J 37: e98726 30061313 10.15252/embj.201798726 PMC6187204

[kiab187-B29] Kinoshita T , Seki M ( 2014) Epigenetic memory for stress response and adaptation in plants. Plant Cell Physiol 55: 1859–1863 25298421 10.1093/pcp/pcu125

[kiab187-B30] Leitch AR , Leitch IJ ( 2008) Genomic plasticity and the diversity of polyploidy plants. Science 320: 481–483 18436776 10.1126/science.1153585

[kiab187-B31] Li AL , Geng SF, Zhang LQ, Liu DC, Mao L ( 2015) Making the bread: insights from newly synthesized allohexaploid wheat. Mol Plant 8: 847–859 25747845 10.1016/j.molp.2015.02.016

[kiab187-B32] Li F , Zheng LD, Chen X, Zhao X, Briggs SD, Du HN ( 2017) Gcn5-mediated *Rph1* acetylation regulates its autophagic degradation under DNA damage stress. Nucleic Acids Res 45: 5183–5197 28334815 10.1093/nar/gkx129 PMC5435933

[kiab187-B34] Li S , Lin YJ, Wang P, Zhang B, Li M, Chen S, Shi R, Tunlaya-Anukit S, Liu X, Wang Z et al (2019) The AREB1 transcription factor influences histone acetylation to regulate drought responses and tolerance in *Populus trichocarpa*. Plant Cell 31: 663–686 30538157 10.1105/tpc.18.00437 PMC6482633

[kiab187-B35] Liu K , Cao J, Yu K, Liu X, Gao Y, Chen Q, Zhang W, Peng H, Du J, Xin M, et al (2019) Wheat TaSPL8 modulates leaf angle through auxin and brassinosteroid signaling. Plant Physiol 181: 179–194 31209125 10.1104/pp.19.00248 PMC6716241

[kiab187-B36] Liu S , Liu S, Wang M, Wei T, Meng C, Wang M, Xia G ( 2014) A wheat SIMILAR TO RCD-ONE gene enhances seedling growth and abiotic stress resistance by modulating redox homeostasis and maintaining genomic integrity. Plant Cell 26: 164–180 24443520 10.1105/tpc.113.118687 PMC3963566

[kiab187-B37] Luo M , Cheng K, Xu Y, Yang S, Wu K ( 2017) Plant responses to abiotic stress regulated by histone deacetylases. Front Plant Sci 8: 2147 29326743 10.3389/fpls.2017.02147 PMC5737090

[kiab187-B38] Ma L , Zhang H, Sun L, Jiao Y, Zhang G, Miao C, Hao F ( 2012) NADPH oxidase AtrbohD and AtrbohF function in ROS-dependent regulation of Na^+^/K^+^ homeostasis in Arabidopsis under salt stress. J Exp Bot 63: 305–317 21984648 10.1093/jxb/err280

[kiab187-B39] Madlung A ( 2013) Polyploidy and its effect on evolutionary success: old questions revisited with new tools. Heredity (Edinb) 110: 99–104 23149459 10.1038/hdy.2012.79 PMC3554449

[kiab187-B40] Marino D , Dunand C, Puppo A, Pauly N ( 2012) A burst of plant NADPH oxidases. Trends Plant Sci 17: 9–15 22037416 10.1016/j.tplants.2011.10.001

[kiab187-B41] Miller G , Suzuki N, Ciftci-Yilmaz S, Mittler R ( 2010) Reactive oxygen species homeostasis and signalling during drought and salinity stresses. Plant Cell Environ 33: 453–467 19712065 10.1111/j.1365-3040.2009.02041.x

[kiab187-B42] Mittler R , Vanderauwera S, Suzuki N, Miller G, Tognetti VB, Vandepoele K, Gollery M, Shulaev V, Van Breusegem F ( 2011) ROS signaling: the new wave? Trends Plant Sci 16: 300–309 21482172 10.1016/j.tplants.2011.03.007

[kiab187-B43] Munns R , James RA, Xu B, Athman A, Conn SJ, Jordans C, Byrt CS, Hare RA, Tyerman SD, Tester M et al (2012) Wheat grain yield on saline soils is improved by an ancestral Na^+^ transporter gene. Nat Biotechnol 30: 360–364 22407351 10.1038/nbt.2120

[kiab187-B45] Ni Z , Kim ED, Ha M, Lackey E, Liu J, Zhang Y, Sun Q, Chen ZJ ( 2009) Altered circadian rhythms regulate growth vigour in hybrids and allopolyploids. Nature 457: 327–331 19029881 10.1038/nature07523 PMC2679702

[kiab187-B46] Nishiyama R , Watanabe Y, Fujita Y, Le DT, Kojima M, Werner T, Vankova R, Yamaguchi-Shinozaki K, Shinozaki K, Kakimoto T, et al (2011). Analysis of cytokinin mutants and regulation of cytokinin metabolic genes reveals important regulatory roles of cytokinins in drought, salt and abscisic acid responses, and abscisic acid biosynthesis. Plant Cell 23: 2169–2183 21719693 10.1105/tpc.111.087395 PMC3160038

[kiab187-B47] Otto SP (2007) The evolutionary consequences of polyploidy. Cell 131: 452–462 17981114 10.1016/j.cell.2007.10.022

[kiab187-B48] Perduns R , Horst-Niessen I, Peterhansel C ( 2015) Photosynthetic genes and genes associated with the C4 trait in maize are characterized by a unique class of highly regulated histone acetylation peaks on upstream promoters. Plant Physiol 168: 1378–1951 1969 26111542 10.1104/pp.15.00934 PMC4528772

[kiab187-B49] Powell JJ , Fitzgerald TL, Stiller J, Berkman PJ, Gardiner DM, Manners JM, Henry RJ, Kazan K ( 2017) The defence-associated transcriptome of hexaploid wheat displays homoeolog expression and induction bias. Plant Biotechnol J 15: 533–543 27735125 10.1111/pbi.12651 PMC5362679

[kiab187-B50] Robinson MD , McCarthy DJ, Smyth GK (2010) edgeR: a bioconductor package for differential expression analysis of digital gene expression data. Bioinformatics 26: 139–140 19910308 10.1093/bioinformatics/btp616 PMC2796818

[kiab187-B51] Sagi M , Fluhr R ( 2006) Production of reactive oxygen species by plant NADPH oxidases. Plant Physiol 141: 336–340 16760484 10.1104/pp.106.078089 PMC1475462

[kiab187-B52] Sako K , Kim JM, Matsui A, Nakamura K, Tanaka M, Kobayashi M, Saito K, Nishino N, Kusano M, Taji T et al (2016) Ky-2, a histone deacetylase inhibitor, enhances high-salinity stress tolerance in *Arabidopsis thaliana*. Plant Cell Physiol 57: 776–783 26657894 10.1093/pcp/pcv199

[kiab187-B53] Shan SH , Gorham J, Forster BP, Wyn Jones RG ( 1987) Salt tolerance in the triticeae: the contribution of the D genome to cation selectivity in hexaploid wheat. J Exp Bot 38: 254–269

[kiab187-B54] Shen Y , Wei W, Zhou DX ( 2015) Histone acetylation enzymes coordinate metabolism and gene expression. Trends Plant Sci 20: 614–621 26440431 10.1016/j.tplants.2015.07.005

[kiab187-B55] Soltis PS , Soltis DE ( 2016) Ancient WGD events as drivers of key innovations in angiosperms. Curr Opin Plant Biol 30: 159–165 27064530 10.1016/j.pbi.2016.03.015

[kiab187-B56] Torres MA , Dangl JL ( 2005). Functions of the respiratory burst oxidase in biotic interactions, abiotic stress and development. Curr Opin Plant Biol 8: 397–403 15939662 10.1016/j.pbi.2005.05.014

[kiab187-B57] Uauy C ( 2017) Wheat genomics comes of age. Curr Opin Plant Biol 36: 142–148 28346895 10.1016/j.pbi.2017.01.007

[kiab187-B58] Ueda M , Matsui A, Tanaka M, Nakamura T, Abe T, Sako K, Sasaki T, Kim JM, Ito A, Nishino N, et al (2017) The distinct roles of class I and II RPD3-like histone deacetylases in salinity stress response. Plant Physiol 175: 1760–1773 29018096 10.1104/pp.17.01332 PMC5717743

[kiab187-B59] Van de Peer Y , Mizrachi E, Marchal K ( 2017) The evolutionary significance of polyploidy. Nat Rev Genet 18: 411–424 28502977 10.1038/nrg.2017.26

[kiab187-B60] Walkowiak S , Gao L, Monat C, Haberer G, Kassa MT, Brinton J, Ramirez-Gonzalez RH, Kolodziej MC, Delorean E, Thambugala D, et al (2020) Multiple wheat genomes reveal global variation in modern breeding. Nature 588: 277–283 33239791 10.1038/s41586-020-2961-x PMC7759465

[kiab187-B61] Wang C , Lu G, Hao Y, Guo H, Guo Y, Zhao J, Cheng H (2017) ABP9, a maize bZIP transcription factor, enhances tolerance to salt and drought in transgenic cotton. Planta 246: 453–469 28474114 10.1007/s00425-017-2704-x

[kiab187-B62] Wang WC , Lin TC, Kieber J, Tsai YC ( 2019). Response regulators 9 and 10 negatively regulate salinity tolerance in rice. Plant Cell Physiol 60: 2549–2563 31359043 10.1093/pcp/pcz149

[kiab187-B63] Waszczak C , Carmody M, Kangasjärvi J ( 2018). Reactive oxygen species in plant signaling. Annu Rev Plant Biol 69: 209–236 29489394 10.1146/annurev-arplant-042817-040322

[kiab187-B64] Wendel JF , Jackson SA, Meyers BC, Wing RA ( 2016). Evolution of plant genome architecture. Genome Biol 17: 37 26926526 10.1186/s13059-016-0908-1 PMC4772531

[kiab187-B65] Wu S , Han B, Jiao Y ( 2020) Genetic contribution of paleopolyploidy to adaptive evolution in angiosperms. Mol Plant 13: 59–71 31678615 10.1016/j.molp.2019.10.012

[kiab187-B66] Yang C , Yang Z, Zhao L, Sun F, Liu B ( 2018) A newly formed hexaploid wheat exhibits immediate higher tolerance to nitrogen-deficiency than its parental lines. BMC Plant Biol 18: 113 29879900 10.1186/s12870-018-1334-1 PMC5992729

[kiab187-B67] Yang C , Zhao L, Zhang H, Yang Z, Wang H, Wen S, Zhang C, Rustgi S, von Wettstein D, Liu B ( 2014) Evolution of physiological responses to salt stress in hexaploid wheat. Proc Natl Acad Sci USA 111: 11882–11887 25074914 10.1073/pnas.1412839111 PMC4136619

[kiab187-B68] Zhang F , Wang Y, Yang Y, Wu H, Wang D, Liu J ( 2007) Involvement of hydrogen peroxide and nitric oxide in salt resistance in the calluses from *Populus euphratica*. Plant Cell Environ 30: 775–785 17547650 10.1111/j.1365-3040.2007.01667.x

[kiab187-B69] Zhang T , Hu Y, Jiang W, Fang L, Guan X, Chen J, Zhang J, Saski CA, Scheffler BE, Stelly DM, et al (2015) Sequencing of allotetraploid cotton (*Gossypium hirsutum* L. acc. TM-1) provides a resource for fiber improvement. Nat Biotechnol 33: 531–537 25893781 10.1038/nbt.3207

[kiab187-B70] Zhang Y , Wang Y, Wen W, Shi Z, Gu Q, Ahammed GJ, Cao K, Shah Jahan M, Shu S, Wang J et al (2020) Hydrogen peroxide mediates spermidine-induced autophagy to alleviate salt stress in cucumber. Autophagy **29**: 1–15 10.1080/15548627.2020.1847797 PMC8525995 33172324

[kiab187-B71] Zheng M , Liu X, Lin J, Liu X, Wang Z, Xin M, Yao Y, Peng H, Zhou DX, Ni Z et al (2019). Histone acetyltransferase GCN5 contributes to cell wall integrity and salt stress tolerance by altering the expression of cellulose synthesis genes. Plant J 97: 587–602 30394596 10.1111/tpj.14144

[kiab187-B72] Zheng Y , Ding Y, Sun X, Xie S, Wang D, Liu X, Su L, Wei W, Pan L, Zhou DX ( 2016) Histone deacetylase HDA9 negatively regulates salt and drought stress responsiveness in Arabidopsis. J Exp Bot 67: 1703–1713 26733691 10.1093/jxb/erv562

[kiab187-B73] Zhou S , Jiang W, Long F, Cheng S, Yang W, Zhao Y, Zhou DX ( 2017) Rice homeodomain protein WOX11 recruits a histone acetyltransferase complex to establish programs of cell proliferation of crown root meristem. Plant Cell 29: 1088–1104 28487409 10.1105/tpc.16.00908 PMC5466029

