# Supplementary material for: Histone acetyltransferase TaHAG1 acts as a crucial regulator to strengthen salt tolerance of hexaploid wheat
Source: Plant Physiol. 2021 Apr 23;186(4):1951–69. doi: 10.1093/plphys/kiab187 (PMC8331135; doi:10.1093/plphys/kiab187)
Supplement: kiab187_Supplementary_Data [file kiab187_supplementary_data.zip › pp.01643.2020-s01.pdf]

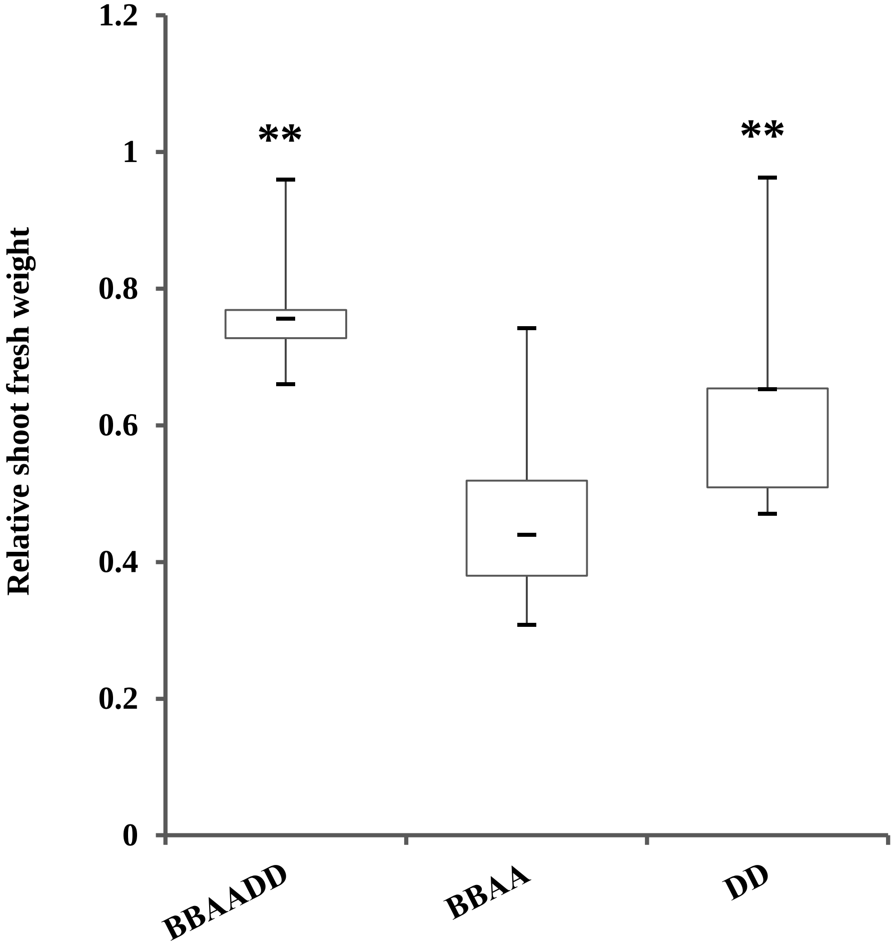

Supplemental Figure S1. Comparative analyses of the relative shoot fresh weight for natural wheat accessions with varying ploidy under salt stress. Box edges represent the 0.25 quantile and 0.75 with the maximum, minimum and average values are represented by bold lines. Differences between the varying ploidy (n = 16) were analyzed by Student's t-test. **P < 0.01.

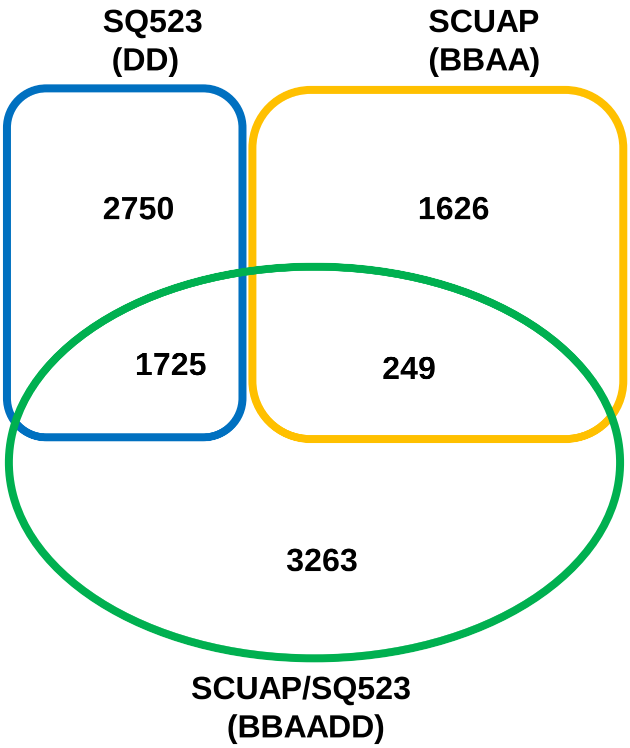

Supplemental Figure S2. Venn diagram showing overlap of upregulated genes between SCAUP/SQ523 with its allotetraploid parent SCAUP or with its diploid parent SQ523 in response to salt stress. The synthetic allohexaploid wheat SCUAP/SQ523 has three closely related, yet distinct genomes (A, B and D) that contain complementary sets of homeologous genes in collinear order across individual chromosomes. The A and B genomes of SCUAP/SQ523 inherited from allotetraploid SCAUP, and D genome inherited from diploid species *Ae. tauschii* SQ523.

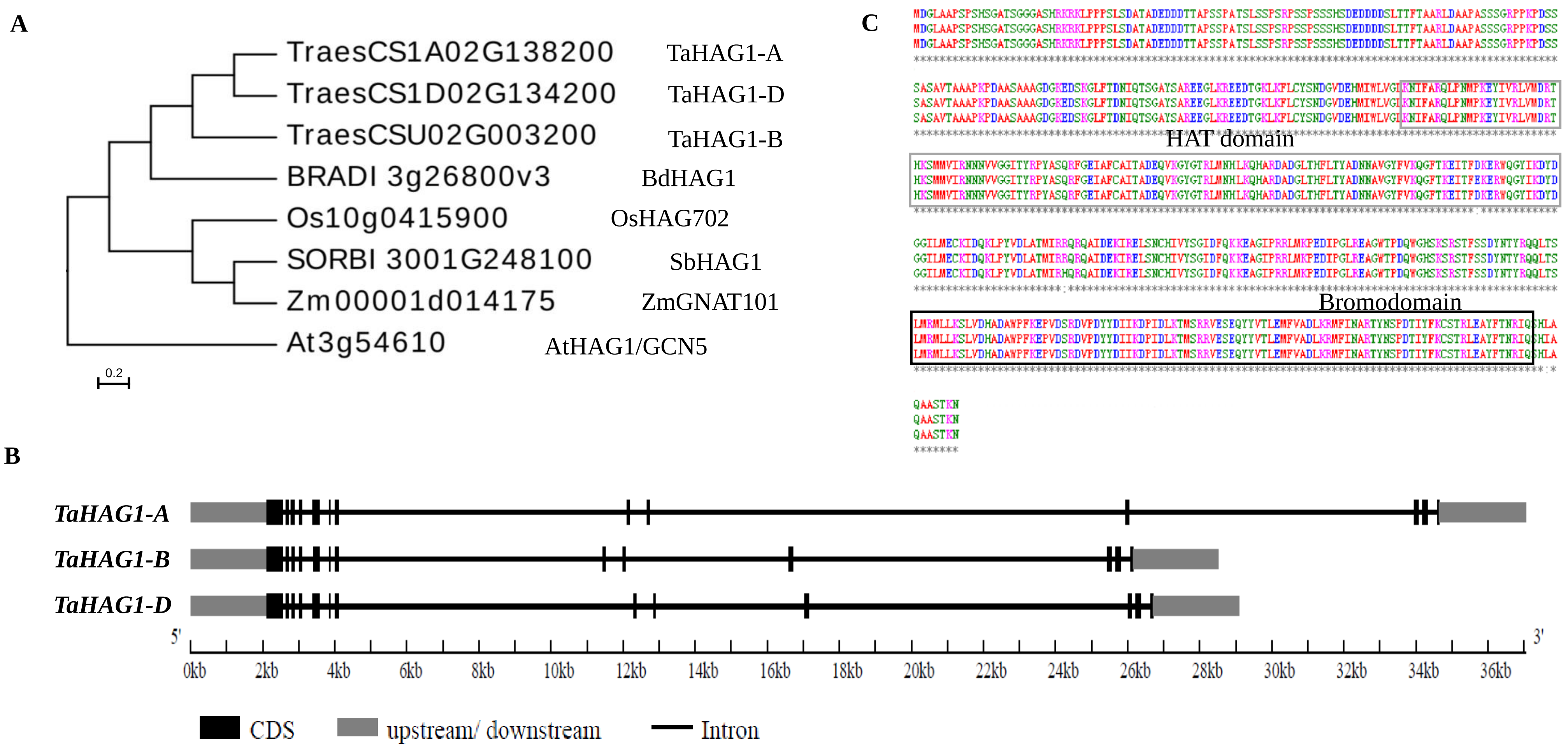

Supplemental Figure S3. Sequence analysis of *TaHAG1* homeologs. A, Phylogenetic relationship of three TaHAG1 homeologs with orthologs in *Brachypodium distachyon*, rice, maize, *sorghum* and *Arabidopsis*. B, Schematic diagram of genomic and their corresponding cDNA sequences of three *TaHAG1* homeologous genes. C, Comparison the amino acids of three TaHAG1 homeologs. Identical amino acids are indicated by asterisks, and gray boxes indicate residues N-terminal HAT domain; black box indicates C-terminal bromodomain.

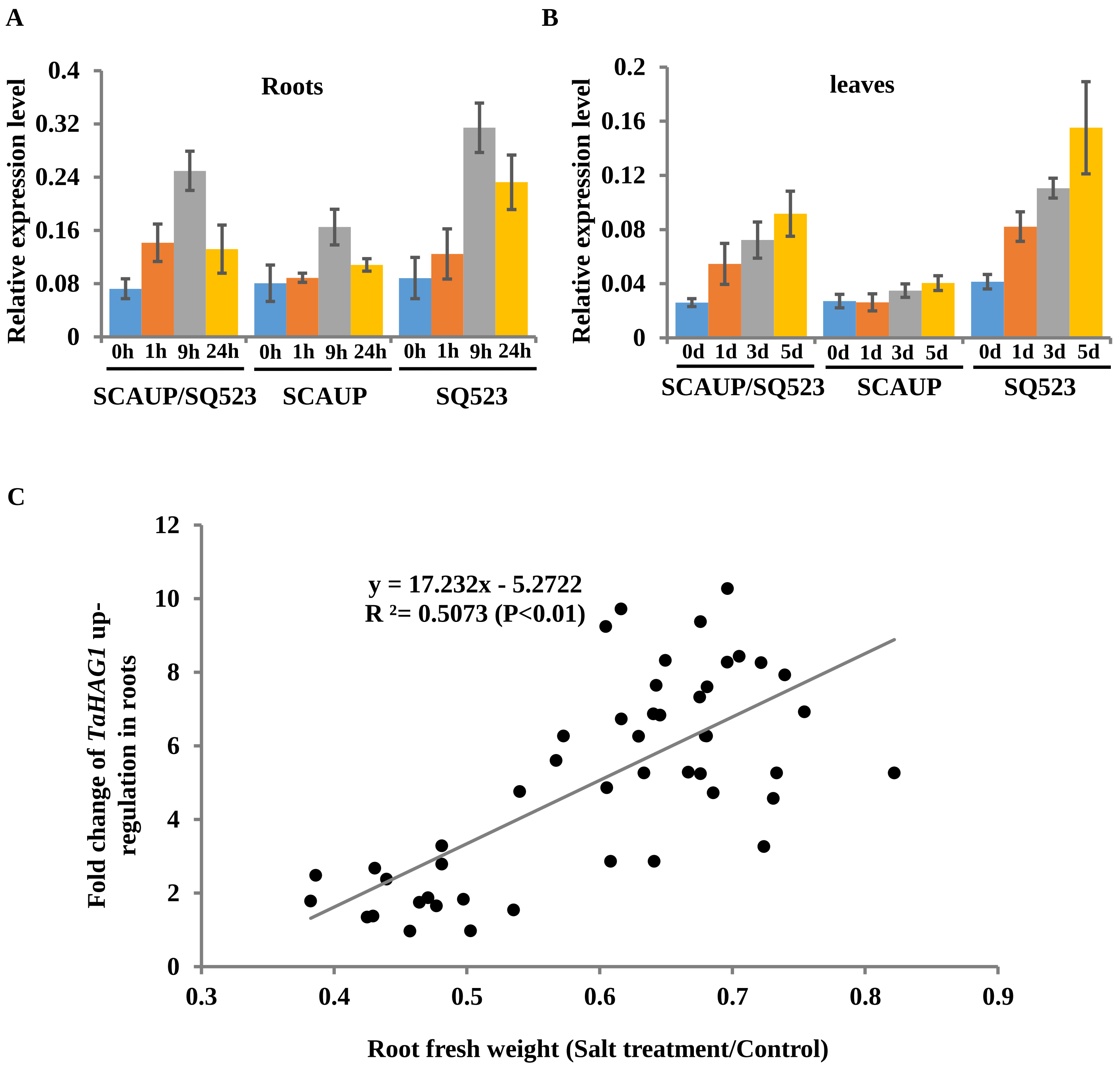

Supplemental Figure S4. Expression pattern of *TaHAG1* in wheat accessions with different ploidy under salt stress. A and B, The expression pattern of *TaHAG1* in roots (A) and leaves (B) of SCAUP/SQ523 and SCAUP, SQ523 under salt stress treatment. The expression of *TUBB3* was used to normalize mRNA levels. The values are means (± SE) of three biological replicates. C, Correlation coefficients between the relative root weight with the expression levels of *TaHAG1* among different ploidy wheat accessions. The x-axis denotes a percentage of NaCl-treated seedlings relative to the same genotype grown on control condition. The y-axis denotes the fold change of *TaHAG1* up-regulation expression in wheat accessions with different ploidy before and after 200 mM NaCl treatment. Statistical significance was determined by ANOVA.

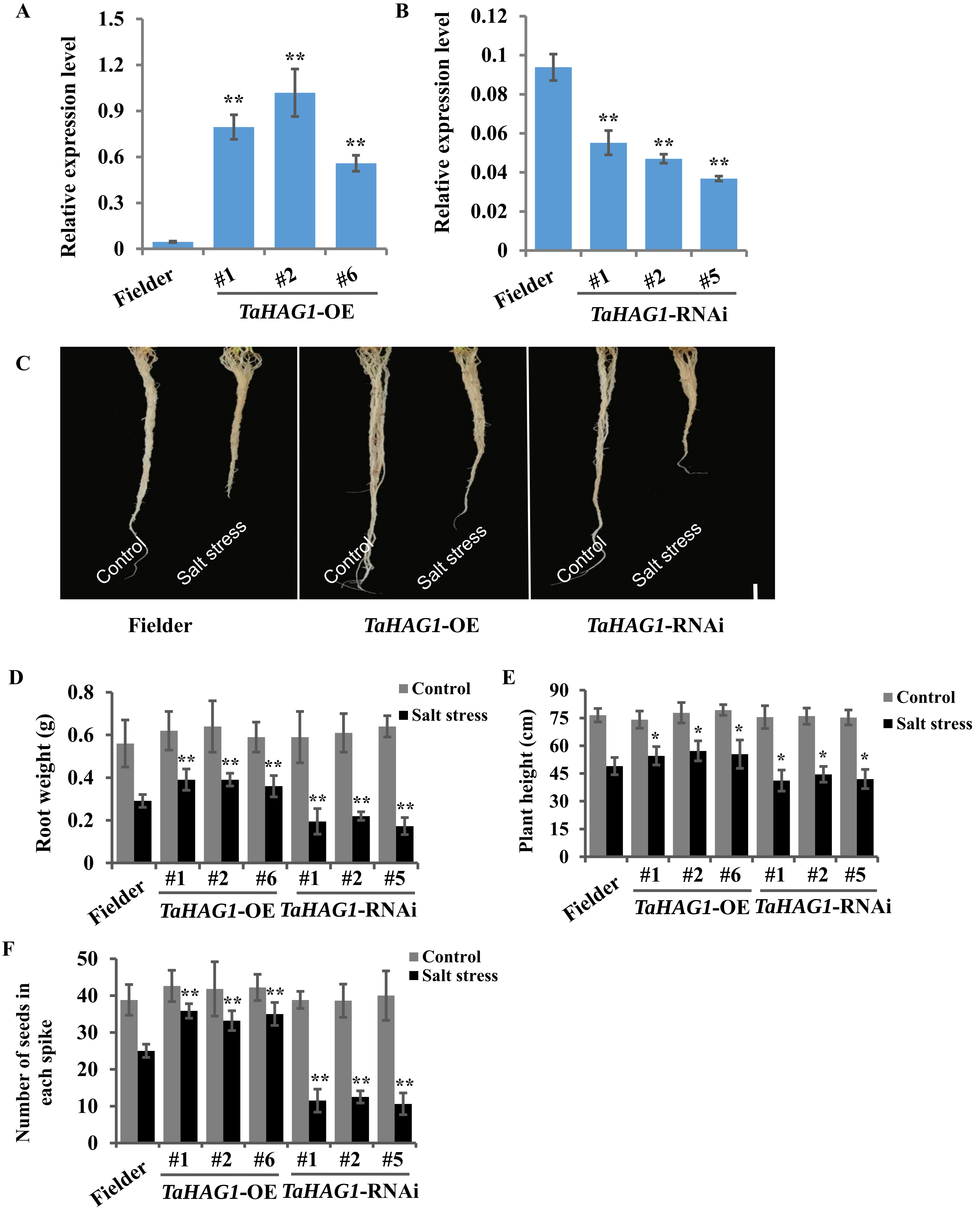

Supplemental Figure S5. Identification of the role of *TaHAG1* in wheat salt tolerance. A and B, The *TaHAG1*-OE (A), *TaHAG1*-RNAi (B) transgenic lines confirmed by RT-qPCR. C, Root phenotype of 4-week-old *TaHAG1*-OE, *TaHAG1*-RNAi lines with wild type Fielder plants under normal conditions or 200 mM NaCl treatment. Scale bars: 5 cm. D, Comparison the root weight of 4-week-old *TaHAG1*-OE, *TaHAG1*-RNAi plants under salt stress conditions. E and F, Statistical data for plant height (E) and number of seeds per spike (F) of the *TaHAG1*-OE, *TaHAG1*-RNAi lines with wild type Fielder plants under salt stress treatments. Mean and SD values were derived from measurements of at least 15 plants of each kind of genotype in three independent experiments. Asterisks indicate significant differences between *TaHAG1* transgenic lines with wild type plants under salt stress conditions (*$P < 0.05$, **$P < 0.01$ by two-sided t-test).

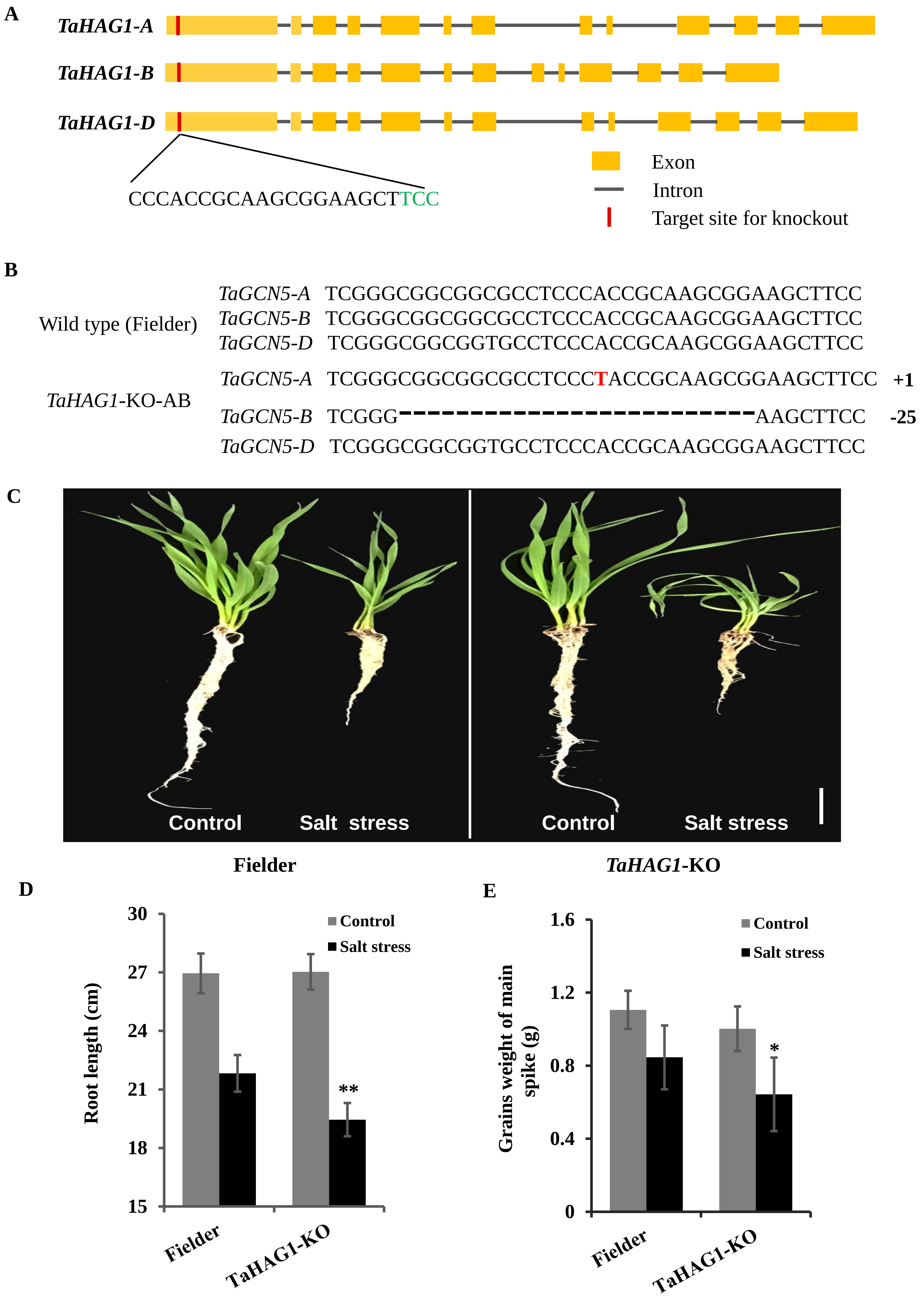

Supplemental Figure S6. Knockout of *TaHAG1* causes reduced salt tolerance in wheat. A, Target site designed for knocking out the *TaHAG1* genes by CRISPR/Cas9 system. B, Verification of the knockout (KO) lines by PCR-based sequencing. The representative transgenic lines (*TaHAG1*-KO-AB) are generated from wheat cultivar Fielder genetic background. Deleted nucleotides are represented 'by '–', and' inserted nucleotides are highlighted in red. The numbers on the right represent the number of nucleotides involved in the indel-created events with '+' or '–'. C and D, Representative pictures (C) and statistical comparisons of root length (D) of 3-week-old *TaHAG1*-KO lines with wild type Fielder plants under normal conditions or 200 mM NaCl treatment. Scale bars: 10 cm. E, Statistical data for grain weight per spike of the *TaHAG1*-KO lines with wild type Fielder plants under salt stress. Mean and SD values were derived from measurements of at least 15 plants of each kind of genotype in three independent experiments. Asterisks indicate significant differences between *TaHAG1*-KO lines with wild type plants under salt stress conditions (*P < 0.05, **P < 0.01 by two-sided t-test).

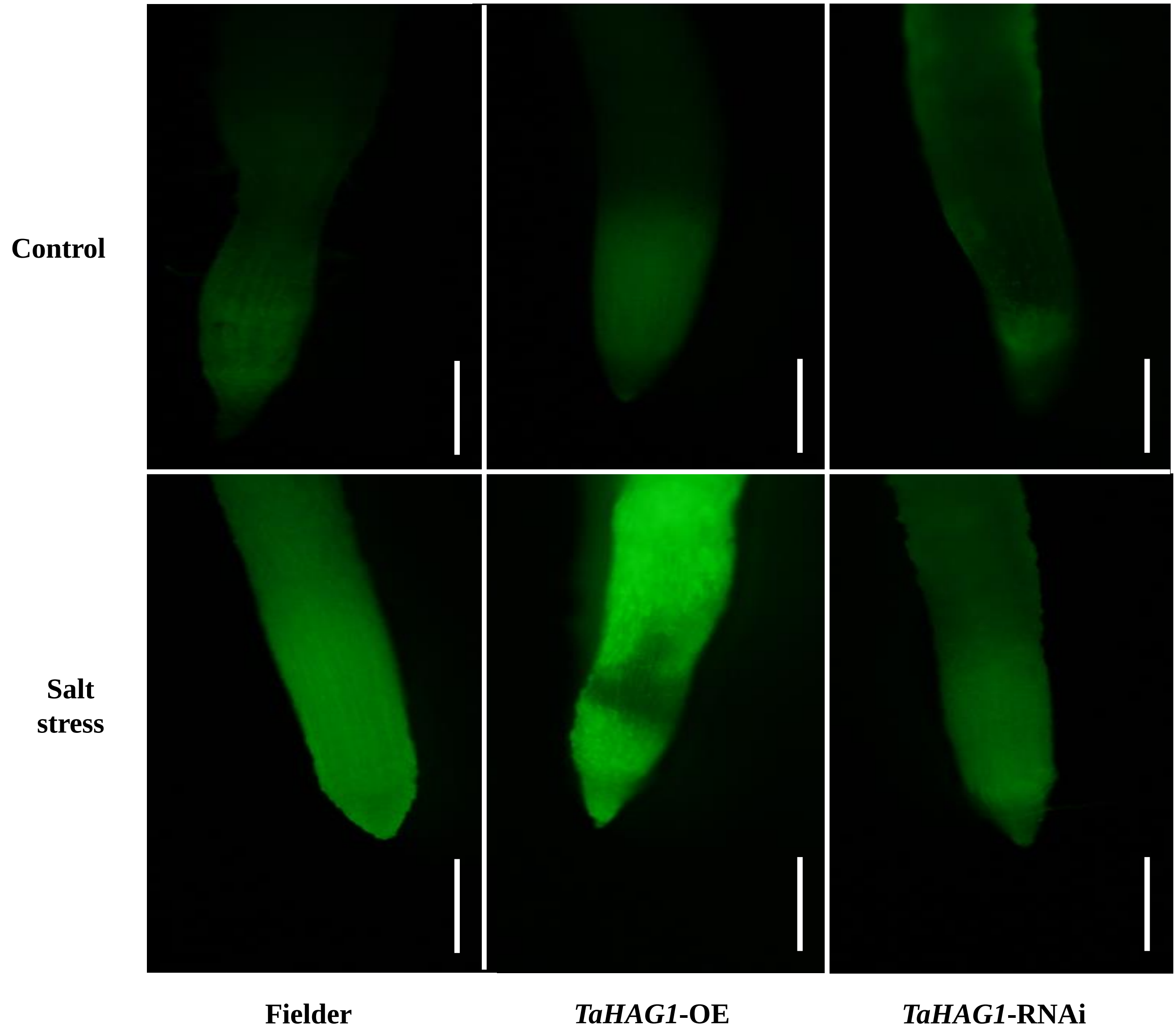

Supplemental Figure S7. ROS production detected by H2DCF-DA fluorescence in roots of the wild type Fielder and *TaHAG1*-OE, *TaHAG1*-RNAi plants under control or salt stress conditions. 7-day-old seedlings grown in a hydroponic culture were treated with 200 mM NaCl for 3 h, then for H2DCFDA labeling assay analysis. Scale bars, 500 μm.

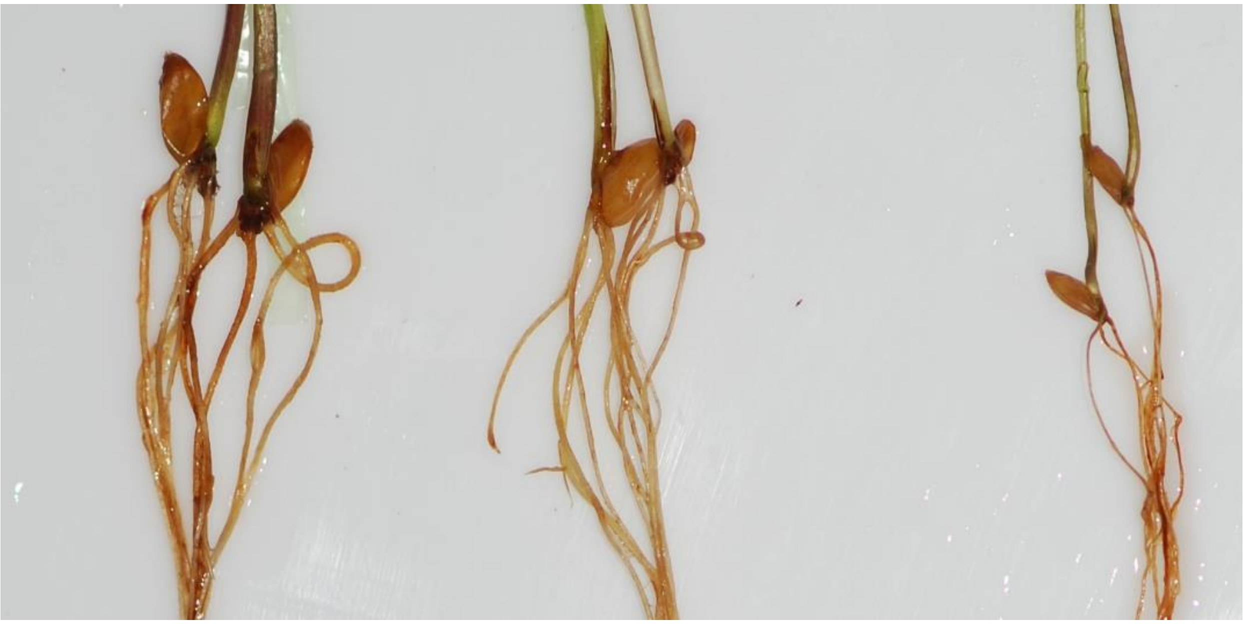

SCAUP/SQ523 (BBAADD) **SCAUP (BBAA)** **SQ523 (DD)**

Supplemental Figure S8. DAB staining of NaCl-induced ROS production in roots of synthetic allohexaploid wheat SCAUP/SQ523, its tetraploid parent, *Tritium turgidum* SCAUP and its diploid parent, *Ae. tauschii* SQ523 under salt stress conditions. Tawny shading indicates accumulation of H_2O_2. Scale bars, 1 cm.

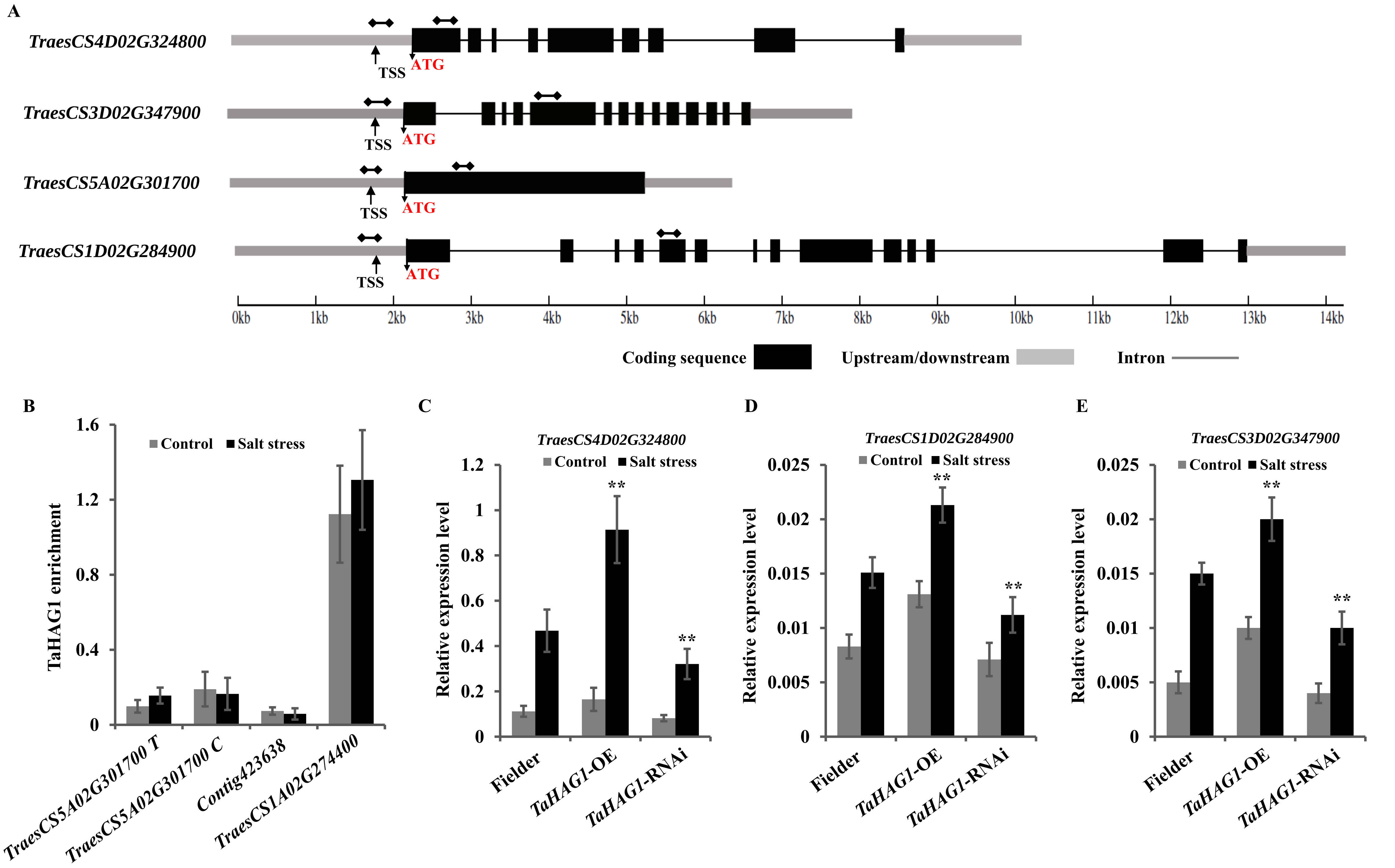

Supplemental Figure S9. TaHAG1 enrichment and expression pattern analysis of the target genes. A, Schematic representation of gene structures indicating regions examined by the ChIP assay. The positions of the primer sets (dumbbell) used in the ChIP assay relative to the transcriptional start sites (TSSs) and ATG codon are shown. B, Analysis of TaHAG1 enrichment in *TraesCS5A02G301700* gene in wheat plants before and after salt stress treatments. T and C indicate TSSs and coding regions, respectively. C to E, Transcript levels of the respiratory burst oxidase homologue genes in leaves of *TaHAG1*-OE, *TaHAG1*-RNAi lines with wild type Fielder plants under normal conditions or salt stress treatment. RT-qPCR results were normalized with the *β-Actin* reference gene. Error bars represent the SEM of three replicates. Similar results were obtained from three independent biological experiments. Asterisks indicate significant differences between *TaHAG1* transgenic lines with wild type plants under salt stress conditions (**P < 0.01 by two-sided t-test).

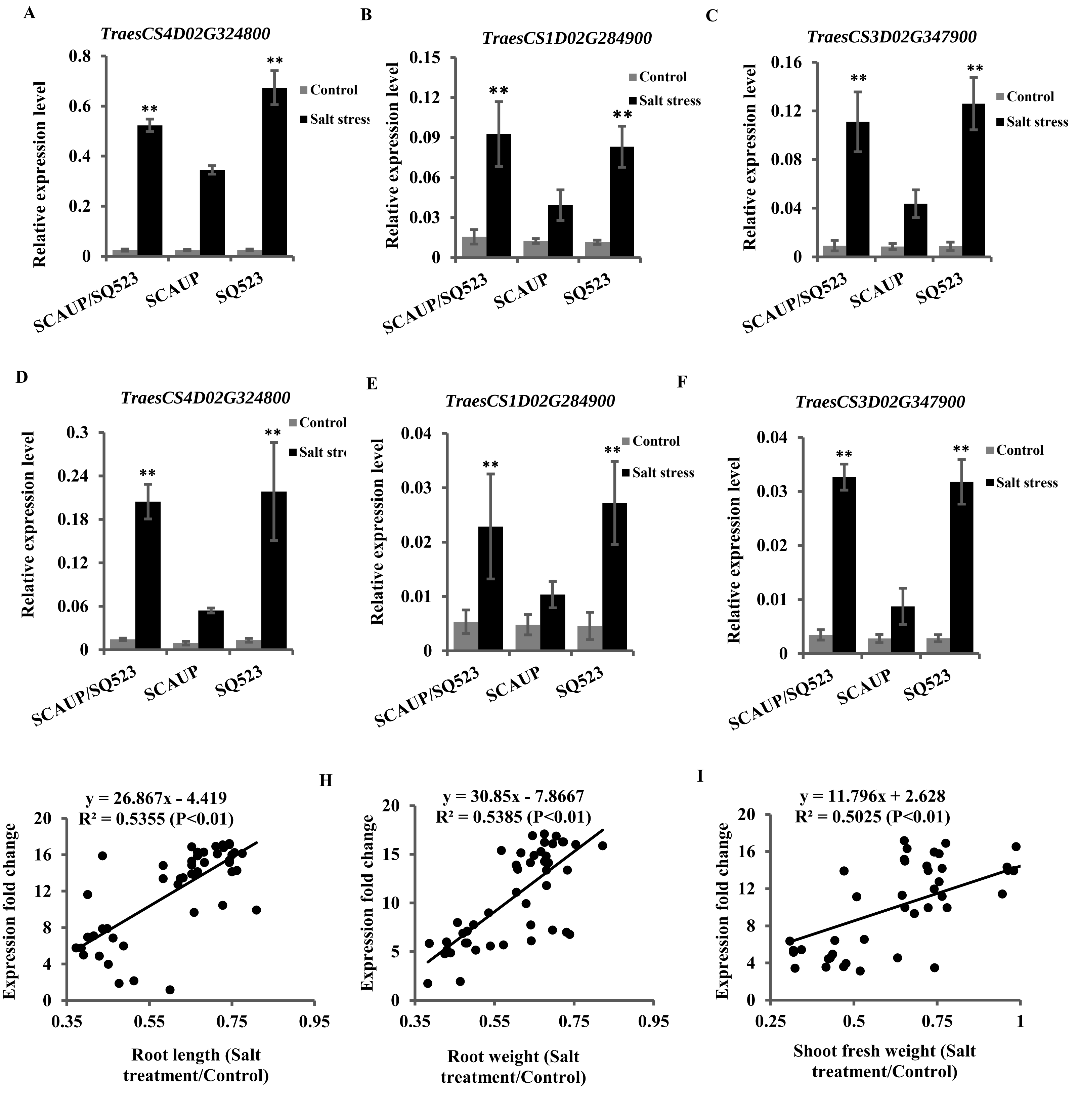

Supplemental Figure S10. Salinity induced respiratory burst oxidase genes expression was correlated with salt tolerance variation in polyploidy wheat. A to F, Expression patterns of three TaHAG1 target genes *TraesCS4D02G324800*, *TraesCS1D02G284900* and *TraesCS3D02G347900* in roots (A to C) and leaves (D to F) of synthetic allopolyploid wheat SCAUP/SQ523 and their parents SCAUP, SQ523 under salt stress. 7-day-old seedlings grown in a hydroponic culture were treated with 200 mM NaCl for 9 h in roots and 48 h in leaves. The expression of *β-Actin* was used to normalize mRNA levels. The values are means (± SE) of three biological replicates. Asterisks indicate significant differences between SCAUP/SQ523, SQ523 plants with SCAUP plants under salt stress conditions (*$P < 0.05$, **$P < 0.01$ by two-sided t-test). G to I, Correlation coefficients between the relative roots length (G), root fresh weight (H) and relative shoot fresh weight (I) with the expression levels of *TraesCS4D02G324800* gene among different ploidy wheat accessions. The x-axis denotes a percentage of NaCl-treated seedlings relative to the same genotype grown on control condition. The y-axis denotes the fold change of *TraesCS4D02G324800* up-regulation expression in wheat accessions with different ploidy before and after 200 mM NaCl treatment. Statistical significance was determined by ANOVA.

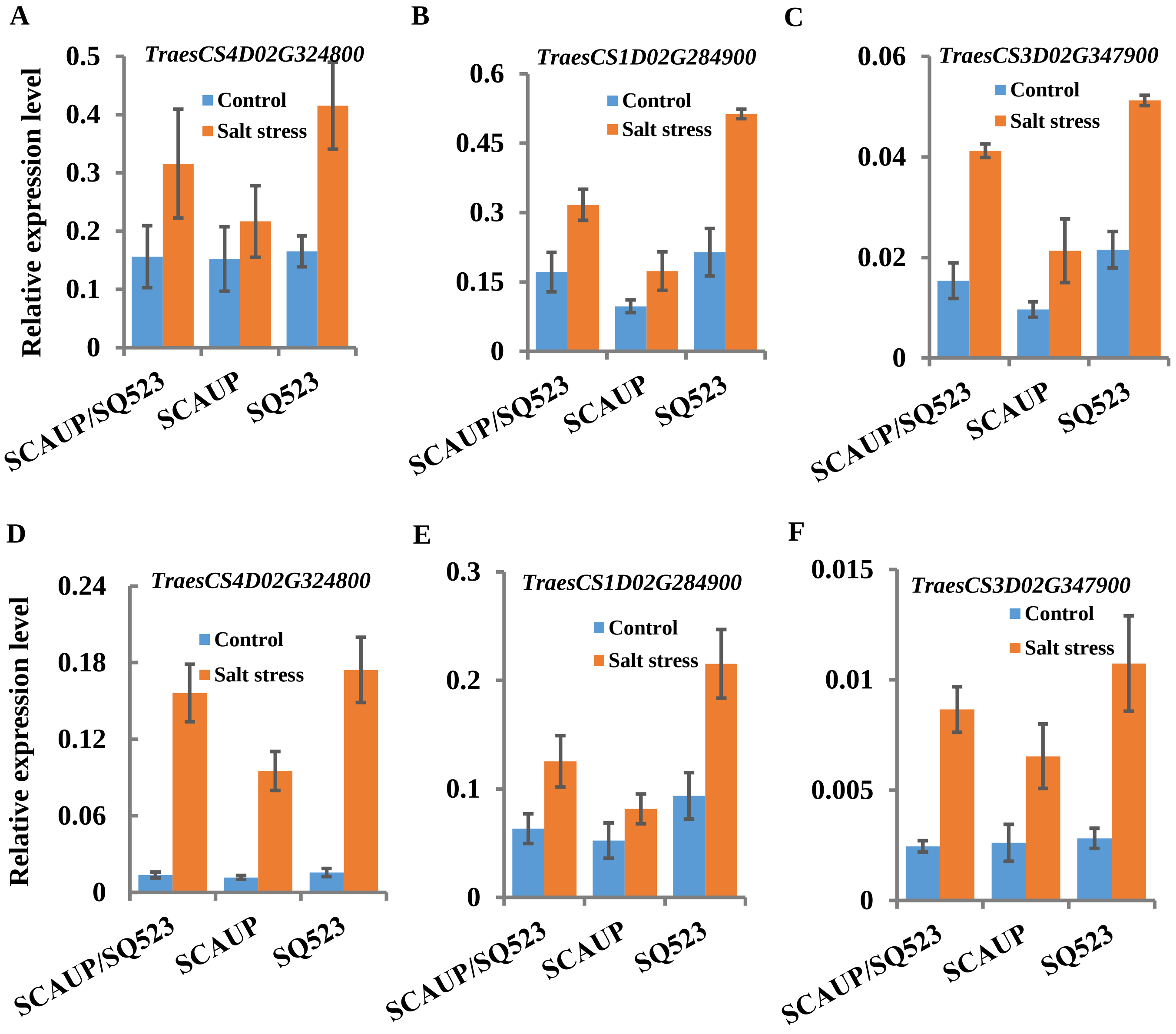

Supplemental Figure S11. Transcript levels of three TaHAG1 target genes in synthetic allopolyploid wheat SCAUP/SQ523 and their parents SCAUP, SQ523 under salt stress treatment. The expression of *TUBB3* was used to normalize mRNA levels. Expression patterns of *TraesCS4D02G324800*, *TraesCS1D02G284900* and *TraesCS3D02G347900* in roots (A to C) and leaves (D to F) of SCAUP/SQ523 and their parents SCAUP, SQ523 under salt stress. 7-day-old seedlings grown in a hydroponic culture were treated with 200 mM NaCl for 9 h in roots and 48 h in leaves. The values are means (± SE) of three biological replicates.

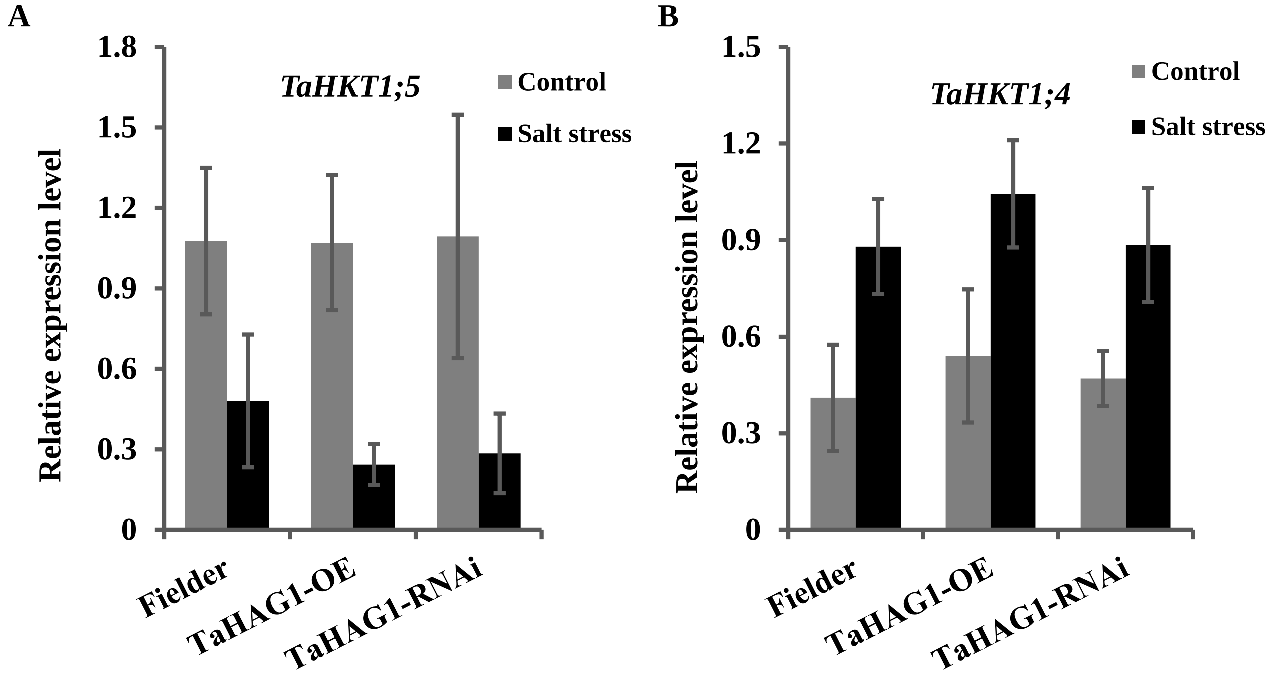

Supplemental Figure S12. Expression pattern of *TaHKT1;5* and *TaHKT1;4* in *TaHAG1* transgenic lines under salt stress. Transcript levels of the *TaHKT1;5* (A) and *TaHKT1;4* (B) genes in roots of *TaHAG1*-OE, *TaHAG1*-RNAi lines with wild type Fielder plants under normal conditions or salt stress treatment. The values are means (± SE) of three biological replicates.

TaHAG1-A	*TaHAG1-B*	*TaHAG1-D*
A-box	AAGAA-motif	A-box
ABRE	ABRE	ABRE
ARE	ARE	ABRE3a
AT~TATA-box	AuxRR-core	ABRE4
Box 4	CAAT-box	ACE
CAAT-box	CCAAT-box	ARE
CAT-box	CGTCA-motif	ATCT-motif
CCAAT-box	DRE core	AT~TATA-box
CCGTCC motif	ERE	CAAT-box
CCGTCC-box	G-Box	CCGTCC motif
CGTCA-motif	G-box	CCGTCC-box
DRE core	I-box	CGTCA-motif
ERE	LTR	G-Box
G-box	MBS	G-box
GATA-motif	MYB	GATA-motif
I-box	MYB recognition site	GCN4_motif
LTR	MYC	I-box
MRE	Myb	LAMP-element
MYB	Myb-binding site	LTR
MYB recognition site	STRE	MYC
MYB-like sequence	Sp1	Myb
MYC	TATA	O2-site
Myb	TATA-box	STRE
Myb-binding site	TGA-element	TATA-box
STRE	TGACG-motif	TC-rich repeats
Sp1	Unnamed__1	TCA
TATA-box	Unnamed__4	TGA-element
TC-rich repeats	WRE3	TGACG-motif
TCCC-motif	as-1	Unnamed__1
TCT-motif	box S	Unnamed__4
TGA-element	circadian	W box
TGACG-motif		as-1
Unnamed__1		
Unnamed__2		
Unnamed__4		
W box		
WRE3		
as-1		
dOCT		

Supplemental Figure S13. The cis-acting regulatory elements analysis for promoter sequences of *TaHAG1-A*, *TaHAG1-B*, *TaHAG1-D* genes from hexaploid wheat genotype Fielder using PlantCARE database. Green shading indicates the *cis*-acting elements that specific in *TaHAG1-D* promoters.
